# Multiomic spatial landscape of innate immune cells at human central nervous system borders

**DOI:** 10.1038/s41591-023-02673-1

**Published:** 2023-12-20

**Authors:** Roman Sankowski, Patrick Süß, Alexander Benkendorff, Chotima Böttcher, Camila Fernandez-Zapata, Chintan Chhatbar, Jonathan Cahueau, Gianni Monaco, Adrià Dalmau Gasull, Ashkan Khavaran, Jürgen Grauvogel, Christian Scheiwe, Mukesch Johannes Shah, Dieter Henrik Heiland, Oliver Schnell, Filiz Markfeld-Erol, Mirjam Kunze, Robert Zeiser, Josef Priller, Marco Prinz

**Affiliations:** 1 Institute of Neuropathology, Faculty of Medicine, University of Freiburg, Freiburg, Germany; 2 Department of Molecular Neurology, Friedrich Alexander University Erlangen-Nürnberg, University Hospital Erlangen, Erlangen, Germany; 3 Neuropsychiatry Unit and Laboratory of Molecular Psychiatry, Charité, Universitätsmedizin Berlin and DZNE, Berlin, Germany; 4 Institute for Transfusion Medicine and Gene Therapy, Medical Center-University of Freiburg, Freiburg, Germany; 5 Department of Neurosurgery, Faculty of Medicine, University of Freiburg, Freiburg, Germany; 6 Department of Gynecology, Obstetrics, and Perinatology, Faculty of Medicine, University Hospital, Freiburg, Germany; 7 Department of Internal Medicine I, Faculty of Medicine, Medical Center-University of Freiburg, Freiburg, Germany; 8 Signalling Research Centres BIOSS and CIBSS, University of Freiburg, Freiburg, Germany; 9 Department of Psychiatry and Psychotherapy, School of Medicine and Health, Technical University of Munich, Munich, Germany; 10 University of Edinburgh and UK DRI, Edinburgh, UK

**Keywords:** Neuroimmunology, Glial biology

## Abstract

The innate immune compartment of the human central nervous system (CNS) is highly diverse and includes several immune-cell populations such as macrophages that are frequent in the brain parenchyma (microglia) and less numerous at the brain interfaces as CNS-associated macrophages (CAMs). Due to their scantiness and particular location, little is known about the presence of temporally and spatially restricted CAM subclasses during development, health and perturbation. Here we combined single-cell RNA sequencing, time-of-flight mass cytometry and single-cell spatial transcriptomics with fate mapping and advanced immunohistochemistry to comprehensively characterize the immune system at human CNS interfaces with over 356,000 analyzed transcriptomes from 102 individuals. We also provide a comprehensive analysis of resident and engrafted myeloid cells in the brains of 15 individuals with peripheral blood stem cell transplantation, revealing compartment-specific engraftment rates across different CNS interfaces. Integrated multiomic and high-resolution spatial transcriptome analysis of anatomically dissected glioblastoma samples shows regionally distinct myeloid cell-type distributions driven by hypoxia. Notably, the glioblastoma-associated hypoxia response was distinct from the physiological hypoxia response in fetal microglia and CAMs. Our results highlight myeloid diversity at the interfaces of the human CNS with the periphery and provide insights into the complexities of the human brain’s immune system.

## Abstract (web summary)

A single-cell spatial transcriptomic and proteomic study of immune cells in the human CNS border compartments reveals differences in CNS-associated macrophages across age, perturbation and disease.

## Main

The CNS interfaces are physical, immunological and molecular barriers ensuring the structural integrity of the CNS parenchyma while facilitating waste disposal^1^. Recent evidence identified CNS interfaces as anatomical sites for pathogen invasion, tumor dissemination and neurodegeneration^2–4^. In line with the diverse immunological functions, CNS interfaces host more diverse immune populations than the CNS parenchyma (PC)^4–6^. The mouse perivascular space (PV), leptomeninges (LM), choroid plexus (CP) and dura mater (DM) contain myeloid cells, lymphoid cells and dendritic cells (DCs), whereas the PC largely contains only microglia^7,8^. Notably, non-parenchymal PV, LM, CP and DM macrophages represent the main immune cells at CNS interfaces^8,9^. Collectively these cells are called CAMs or border-associated macrophages (BAMs)^10–15^. Despite their important functions^11,16^, their complexity in human brains remains unexplored.

For many years, it was thought that all CAMs originated from short-lived bone-marrow-derived monocytes that are continuously replaced postnatally^17^. Recent evidence established a common prenatal yolk sac-derived origin of CAMs^9,18–20^. CAMs and microglia were shown to be long-lived and self-renewing^9,21^. Despite these common origins, adult microglia and CAMs show distinct transcriptional phenotypes. Namely, mouse microglia mainly express *Hexb*, *Tmem119* and *P2ry12*, whereas CAMs characteristically show *Mrc1*, *Lyve1* and *Ms4a7* expression^7,8,10,22,23^.

Recently, we described that while microglia and LM CAMs are present prenatally, PV CAMs only arose postnatally following the establishment of the PV space^24^. Notably, direct cell–cell interactions between CAMs and smooth muscle cells was crucial for the postnatal colonization of the PV space. These findings point to a pronounced integration of CAMs within their niches and major developmental plasticity.

While individual human CNS interface specimens have been initially profiled, an integrated side-by-side transcriptomic, proteomic and cross-species comparisons have only been conducted for the brain PC^25^. Also, a spatially resolved transcriptomic analysis of the pathophysiologically important CAM niche is still lacking. Furthermore, little is known about the development, fates and turnover of human CAMs. Here, we applied single-cell RNA sequencing (scRNA-seq), cellular indexing of transcriptomes and epitopes by sequencing (CITE-seq), mass cytometry and high-resolution spatial transcriptomics to generate a comprehensive molecular census of the immune compartment at the human CNS interfaces during fetal development, adulthood and pathology.

## Results

### Homeostatic immune-cell diversity at human CNS interfaces

**Fig. 1:**
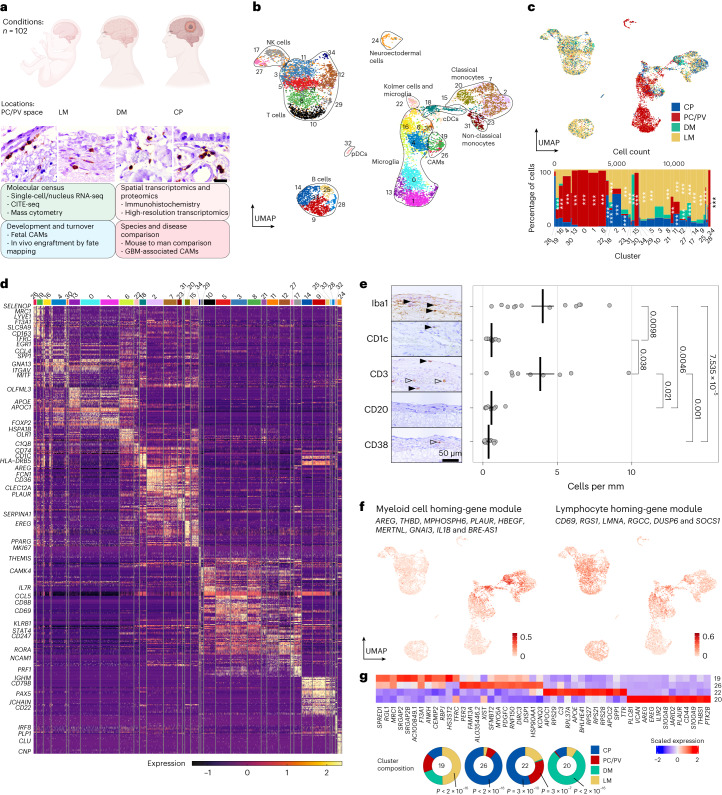
Molecular census of immune cells in human CNS border regions under homeostasis. **a**, Schematic illustration of the present study, including representative CD45^+^ immunohistochemistry images of different CNS interfaces. Scale bar, 20 µm. An overview of biological replicates is provided in Supplementary Table 20. GBM, glioblastoma. **b**, Uniform manifold approximation and projection (UMAP) visualization of 11,166 FACS-sorted CD45^+^ cells from the PC/PV space (*n* = 3,860), LM (*n* = 5,039), CP (*n* = 1,597) and DM (*n* = 670). Color coding and numbers indicate the different clusters. NK, natural killer; pDC, plasmacytoid DC. **c**, UMAP (top) and Marimekko chart (bottom) color coded for the compartment of each. Statistical testing was conducted using one-sided hypergeometric tests with Benjamini–Hochberg multiple-testing adjustment. *P < 0.05; **P < 0.01; ***P < 0.001. The exact *P* values are found in Supplementary Table 2. Significance asterisks are only shown until cluster 24 as clusters 25–31 were relatively small. **d**, Single-cell heat map depicting the expression of the top 20 cluster markers with selected genes shown on the left. Color coding is consistent with **b**. The color scale represents Pearson’s residuals from a regularized negative-binomial regression. **e**, Histological validation of tissue-residency of immune cells in the LM with representative CD45^+^ images. Empty arrowheads indicate intravascular areas, whereas filled arrowheads indicate tissue-resident cells. The dot plot shows quantifications of positive cells per mm of the LM with each dot representing a patient. Between *n* = 9 and *n* = 12 independent patients were assessed per marker. The crossbar indicates the mean counts per mm and the error bar indicates the s.e.m. Statistical testing was performed using a Kruskal–Wallis test followed by Dunn’s test for pairwise multiple comparisons with Holm–Bonferroni adjustment for multiple testing. *P < 0.05; **P < 0.01; ***P < 0.001. **f**, UMAP color coded for the expression of published myeloid (left) and lymphoid (right) homing-gene module scores^29^. The color coding represents module enrichment scores for each cell using the Mann–Whitney *U* statistic. **g**, Heat map of the average expression of the top 12 markers for clusters 19, 26, 22 and 20. The color scale indicates the *z* score. The donut plots show the compartment distribution across clusters. *P* values were calculated using one-sided hypergeometric tests with Benjamini–Hochberg adjustment for multiple testing. Source data

**Extended Data Fig. 1:**
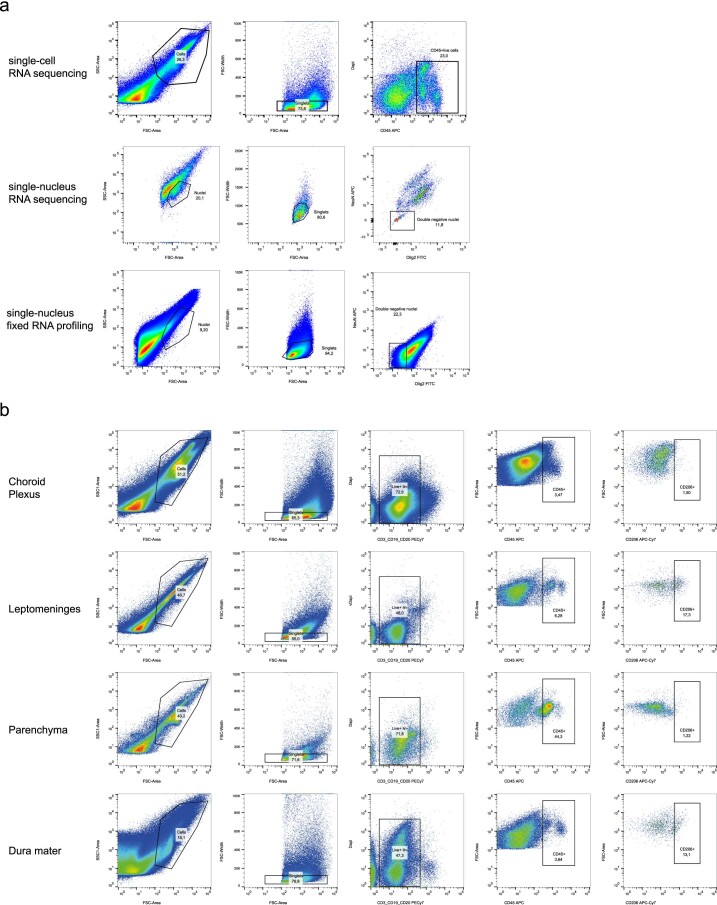
FACS gating strategies. **a**. Representative FACS plots of cell suspensions for 10x single-cell analysis (top figure panel). Cells were gated i. on singlets followed by ii. collection of DAPI- and CD45+ cells. The middle figure panel shows the gating strategy for single-nucleus RNA sequencing. The bottom figure panel shows the gating strategy for single-nucleus fixed RNA profiling. Cells were sorted into 1.5 ml tubes. **b**. For the enrichment of CD206+ cells, i. singlets were selected followed by ii. exclusion of DAPI + CD3 + CD19 + CD20+ cells. iii-iv-. CD45 + CD206+ cells were sorted into 384-well plates.

**Extended Data Fig. 2:**
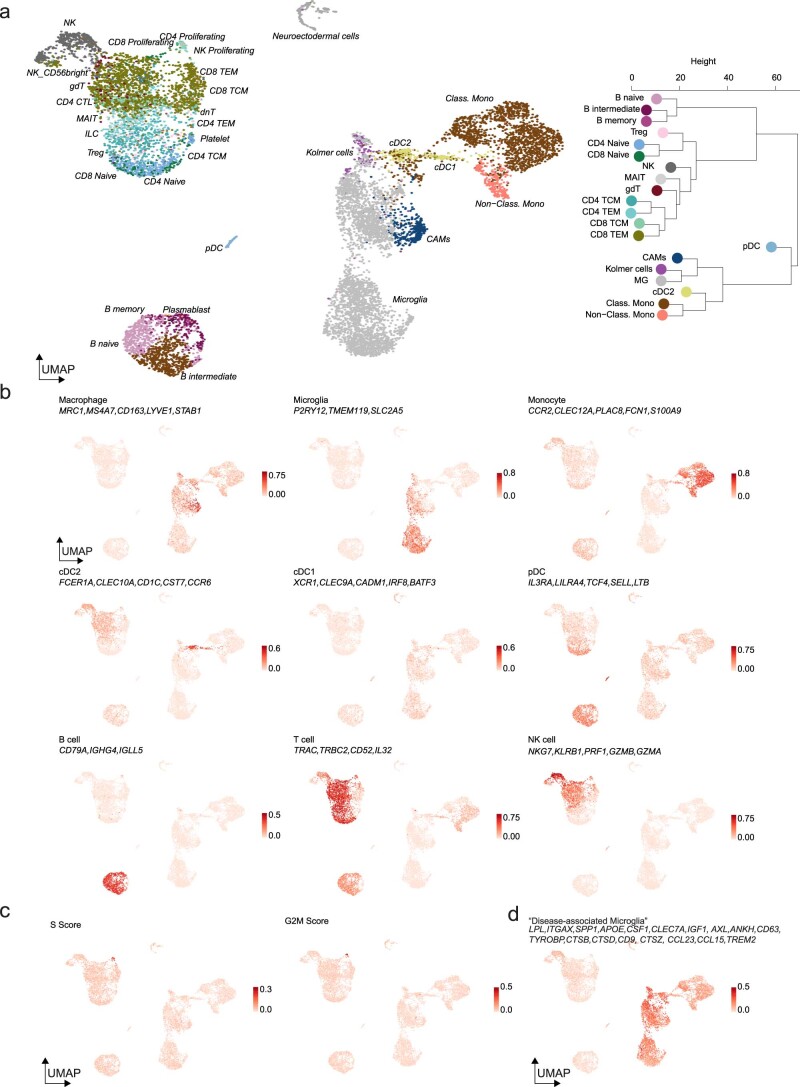
Overview of cell type-enriched gene expression signatures and cell cycle scores. **a**. Cell type assignment of the dataset using the Azimuth human PBMC reference dataset. **b**. UMAP visualizations color coded for the expression of gene modules enriched in the indicated cell types. The color coding represents gene module expression scores for each cell using the Mann-Whitney U statistic. **c**. UMAP visualizations color coded for cell cycle scoring. The color coding represents gene module expression scores for each cell using the Mann-Whitney U statistic. The module score classification is based on published gene expression signatures^27^.**d**. UMAP visualizations color coded for the expression of the published gene module^29^. The color coding represents module enrichment scores for each cell using the Mann-Whitney U statistic. Source data

We profiled the immune compartment at the human CNS interfaces, with state-of-the-art molecular techniques (Fig. 1a). A total of 11,166 CD45^+^ cells from human PC/PV space, LM, CP and DM were enriched by fluorescence-activated cell sorting (FACS) and analyzed using droplet-based scRNA-seq (Extended Data Fig. 1a,b). Cell types were classified using a combination of Azimuth^26^ and published CAM and microglia gene sets^7,8,27^. We found diverse myeloid subsets, including CAMs, microglia, DCs and monocytes, and several lymphoid subsets, including CD4^+^, CD8^+^ and proliferating T lymphocytes^28^ (Fig. 1b, Extended Data Fig. 2a–c and Supplementary Table 1). CAMs (C19 and C26) mostly consisted of CP, LM and DM-derived cells (Fig. 1c,d, and Supplementary Tables 2 and 3). In line with evidence from mice^8,27^, Kolmer cells co-clustered with activated microglia in C22 (Fig. 1c and Supplementary Table 2). Thus, our dataset contained diverse myeloid and lymphoid subsets.

A high degree of vascularization of the CNS interfaces makes the distinction between intravascular and tissue-resident cells challenging. Hence, we conducted histological validation for the main cell-type markers in the LM, the interface predominately included in this study. We detected cells expressing the pan-myeloid marker Iba1 and the T cell marker CD3 outside of blood vessels in considerable numbers (Fig. 1e). Previous work has identified homing markers for myeloid (for example *AREG* and *PLAUR*) and lymphoid cells (for example *CD69*)^29^. We therefore assessed the expression of these gene modules in our data. Notably, the myeloid-homing module was elevated in DM-associated monocytes (C20) (Fig. 1c,f). This corroborates evidence of continuous monocyte-derived macrophage (MoMΦ) engraftment in the murine DM^8^. The lymphocyte-homing module was elevated in T cells of C8 and C21 (Fig. 1f). This analysis confirmed the presence of diverse tissue-resident immune cells in the LM.

Next, cluster marker analysis of the different myeloid populations found distinct markers for CAMs (C19 and C26: *MRC1* and *F13A1*), Kolmer cells (C22: *BHLHE41*, *APOE*, *SPP1* and *TTR*) and the DM-associated MoMΦ (C20: *AREG*, *PLAUR* and *CD44*) (Fig. 1g and Extended Data Fig. 2d). Notably, *APOE* and *SPP1* in Kolmer cells represent so-called ‘disease-associated microglia’ (DAM) markers^8,30^. In summary, we detected a broad transcriptional overlap of CAMs markers across the analyzed human CNS interfaces. Additionally, Kolmer cells showed a microglia-like signature and DM-associated MoMΦ-expressed myeloid-homing genes.

### Comparison of gene signatures in murine and human CAMs

**Extended Data Fig. 3:**
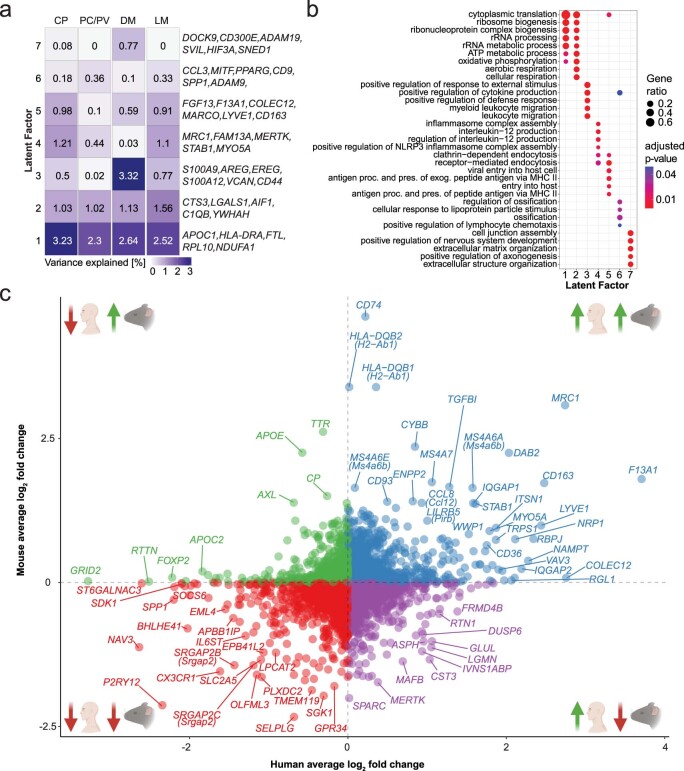
Conserved and distinct signatures of tissue resident myeloid cells in CNS interfaces between mouse and man. **a**. Heatmap visualization of latent factors determined by multiomic factor analysis (MOFA)2 analysis of macrophages across the indicated compartments. The color scale indicates the percentage of variance explained across the compartments. For enhanced readability, the respective values are indicated in each heat map tile. Selected top genes for each latent factor are presented to the right of the heat map. PC/PV: parenchyma/perivascular space, CP: choroid plexus, LM: leptomeninges, DM: dura mater. **b**. Dotplot showing gene ontology term enrichment analysis across the top 100 differentially enriched genes in the MOFA2 latent factors from panel a. The dot size indicates the gene ratio of genes differentially expressed in each cluster over the genes in the indicated gene ontology terms. The color coding of the dot indicates the Benjamini–Hochberg adjusted p value based on a one-sided Fisher’s exact test.**c**. Dot plot depicting the cross-species comparison of differentially expressed orthologue genes between CAMs and microglia in humans and mice. On the x-axis, adjusted average log2-fold changes from the comparison of murine MHC-II^low^ and MHC-II^high^ are signed positively if they are upregulated in the former and negatively if they are upregulated in the latter. The y-axis contains the adjusted average log2-fold changes of the human counterparts. The log2-fold changes were calculated using two-sided unpaired Wilcoxon Rank-Sum tests followed by Bonferroni correction for multiple testing. Orthologue genes in the top right and bottom left quadrants are differentially expressed in the same direction. Orthologue genes in the top left and bottom right quadrants are showing opposite directions of differential expression. Orthologue genes positioned along the axes are only upregulated in one species. The color coding indicates the directionality of the gene expression across species. Blue genes were upregulated in mice and humans; red genes were downregulated in mice and humans; green genes were upregulated in humans and downregulated in mice; violet genes were differentially regulated in humans, but not in mice; orange genes were differentially regulated in mice but not in humans. Source data

Next, we investigated the main factors distinguishing the analyzed anatomical compartments. Multifactorial factor analysis of 4,096 MΦ from 22 samples using MOFA2 (ref. ^31^) identified seven latent factors (Extended Data Fig. 3a and Supplementary Table 4). While factors 1 and 2 were evenly distributed across all compartments, factors 3 and 7 were over-represented in DM. Gene Ontology analysis identified enrichment of the term leukocyte migration in factor 3 and extracellular matrix organization in factor 7 (Extended Data Fig. 3b). Factors 4 and 5 contained macrophage-enriched genes as top genes, including *MRC1*, *STAB1* and *LYVE1* (Extended Data Fig. 3a). Notably, factor 4 was over-represented in CP, PC/PV and LM, whereas factor 5 was over-represented in CP, DM and LM. Gene Ontology analysis identified enrichment of major histocompatibility complex (MHC) II-associated terms in latent factor 5 suggesting higher immune activation in CP, LM and DM but not PC/PV (Extended Data Fig. 3b). In summary, PC/PV-derived myeloid cells show an attenuated expression of antigen-presentation-associated genes.

Species comparison between human CAMs and microglia with a published mouse dataset^7^ showed conserved expression of *MRC1*, *F13A1*, *STAB1* and CD163 in CAMs across species (Extended Data Fig. 3c and Supplementary Table 5). Microglia displayed conserved expression of *P2RY12*, *SLC2A5*, *SELPLG* and *SPP1*. Divergently regulated genes included *APOE* and *AXL* that seemed upregulated in murine CAMs and in human microglia. Conversely, *MAFB* and *MERTK* seemed upregulated in human CAMs and murine microglia. In summary, CAMs and microglia expressed evolutionary conserved gene sets with notable differences between mice and humans.

### Multiomics reveal diversity of steady-state human CAMs

For further validation, we combined three high-dimensional technologies, scRNA-seq, CITE-seq and time-of-flight mass cytometry. The combination of scRNA-seq and mass cytometry previously enabled us to identify and characterize a hitherto unappreciated spectrum of human microglial states^32,33^.

**Fig. 2:**
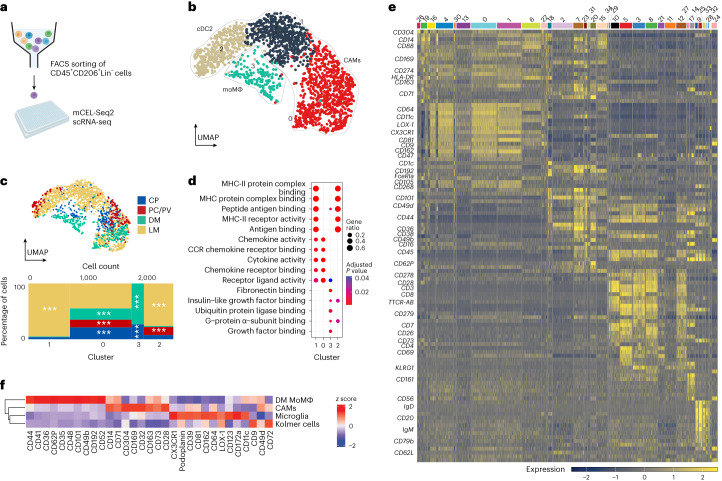
Characterization of human CAMs during homeostasis by single-cell sequencing and CITE-seq. **a**, Schematic representation of the validation experiment presented in **b**–**d**. **b**, UMAP visualization of 1,962 FACS-sorted human CD45^+^CD206^+^Lin^−^ cells color coded for the results of graph-based clustering with Seurat v.4. The indicated cell-type classification was based on a combination of human peripheral blood mononuclear cells^26^ and published marker gene sets^32,44^. **c**, Top: UMAP visualization of the cells from **b**, color coded based on the compartment that the cells were derived from. Bottom: Marimekko chart of the distribution of the compartments per cluster. Asterisks indicate the results of statistical testing using one-sided hypergeometric tests. Adjustment for multiple testing was performed using the Benjamini–Hochberg method. ***P < 0.001. The exact *P* values are in Supplementary Table 6. The color coding is consistent across both panels. **d**, Dot plot showing cluster-wise Gene Ontology term enrichment analysis across the differentially enriched genes in the clusters from **b**. The dot size indicates the gene ratio of genes differentially expressed in each cluster over the genes in the indicated Gene Ontology terms. The color coding of the dot encodes the Benjamini–Hochberg-adjusted *P* value from a one-sided Fisher’s exact test. **e**, Single-cell protein heat map from the top differentially expressed protein markers co-registered with cell transcriptomes presented in Fig. 1. The color scale represents Pearson’s residuals from a regularized negative-binomial regression. **f**, Heat map representation of the average expression of up to ten top differentially expressed surface markers in the indicated cell types. The color scale indicates the *z* score. The dendrogram represents the hierarchical clustering based on the Euclidean distance metric. Source data

**Extended Data Fig. 4:**
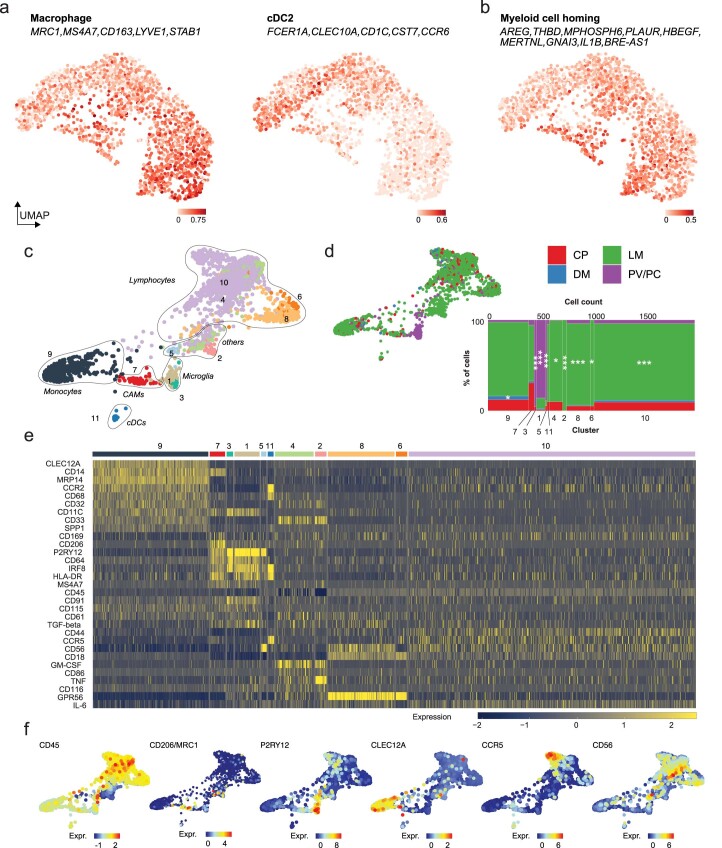
Cell type gene module expression of mCEL-Seq2 data and validation with mass cytometry. **a**. UMAP visualizations color coded for the expression of gene modules enriched in the indicated cell types. The color coding represents module enrichment scores for each cell using the Mann-Whitney U statistic. **b**. UMAP visualization color coded for the expression of published myeloid (left) and lymphoid cell homing gene modules^28^. The color coding represents module enrichment scores for each cell using the Mann-Whitney U statistic. **c**. Spider visualization of 1,999 CD45+ cells analyzed using mass cytometry. The plots are color coded for K-nearest-neighbor density-based X-shift algorithm-based cluster assignment. The indicated cell type assignment is based on the expression of published cell type markers^31^. The cells labeled as ‘others’ did not show CD45 expression. Numbers indicate the different clusters. **d**. Spider presentation of the cells from panel f color coded for the compartment the cells were extracted from. The right bottom panel is showing a Marimekko chart of the distribution of compartments per cluster. CP: choroid plexus, LM: Leptomeninges, PV/PC: perivascular space and parenchyma. **e**. Spider visualization of the cells from panel f color coded for the expression of selected genes. The color scale represents Pearson’s residuals from a regularized negative-binomial regression. **f**. Single-cell heatmap of the protein expression in each cluster depicted in panel f. The color scale represents Pearson’s residuals from a regularized negative-binomial regression. Source data

First, we enriched CAMs for deeper characterization using CD206 (encoded by *MRC1*), a conserved pan-CAM marker. Then, 1,962 CD45^+^CD206^+^CD3^−^CD19^−^CD20^−^ cells from 12 patients were FACS-sorted into multiwell plates and analyzed using the high-sensitivity mCEL-Seq2 protocol^34^ (Fig. 2a). The dataset consisted of CAMs, type 2 conventional DCs (cDC2) and MoMΦ (Fig. 2b and Extended Data Fig. 4a,b). CAMs (C0 and C1) were distributed across all analyzed compartments with enrichment of C0 in PC/PV, CP and DM, and C1 in LM (Fig. 2c and Supplementary Table 6). cDCs (C2) were enriched in LM and PC/PV, whereas MoMΦ (C3) were enriched in CP and DM, underscoring continuous myeloid-derived engraftment^8^. Gene Ontology enrichment analysis showed MHC-II-associated terms in CAMs and cDC2, chemokine and cytokine activity terms in CAMs and fibronectin binding in MoMΦ (Fig. 2d and Supplementary Table 7). In summary, we found a transcriptional spectrum of MHC-II^low^ in C0 and MHC-II^high^ CAMs in C1. These analyses confirmed CD206 protein expression in CAMs, cDC2 and MoMΦ.

Next, we assessed broader surface marker profiles of the immune cells analyzed in Fig. 1 using CITE-seq with 134 cell surface markers. After quality control, we obtained transcriptional and proteomic profiles for 8,161 cells. CAMs (C26 and C19) showed CD304 (encoded by *NRP1*), CD88 (encoded by *C5AR1*) and CD169 (encoded by *SIGLEC1*) as markers (Fig. 2e and Supplementary Table 8). Differential protein expression analysis between the distinct myeloid cell types found separate markers for DM-associated MoMΦ (CD44, CD41 (encoded by *ITGA2B*) and CD36), CAMs (CD71 (encoded by *TFRC*), CD304 (encoded by *NRP1*) and CD169 (encoded by *SIGLEC1*)), microglia (CX3CR1, podoplanin (encoded by *PDPN*) and CD39 (encoded by *ENTPD1*)) and Kolmer cells (CD9, CD49d (encoded by *ITGA4*) and CD72 (Fig. 2f). Additionally, mass cytometry confirmed and expanded protein markers for monocytes (C9: CLEC12A), CAMs (C7: CD206), microglia (C1 and C3: P2RY12) and cDCs (C11: CCR5 and IRF8) (Extended Data Fig. 4c–f and Supplementary Table 9). Thus, our findings provide a combined assessment of gene and protein expression in human CAMs.

### Spatial organization of human CAMs

Next, we examined CAMs in their anatomical niches using multifluorescence confocal microscopy and spatial transcriptomics through in situ sequencing (ISS) and the Nanostring CosMx technology.

**Fig. 3:**
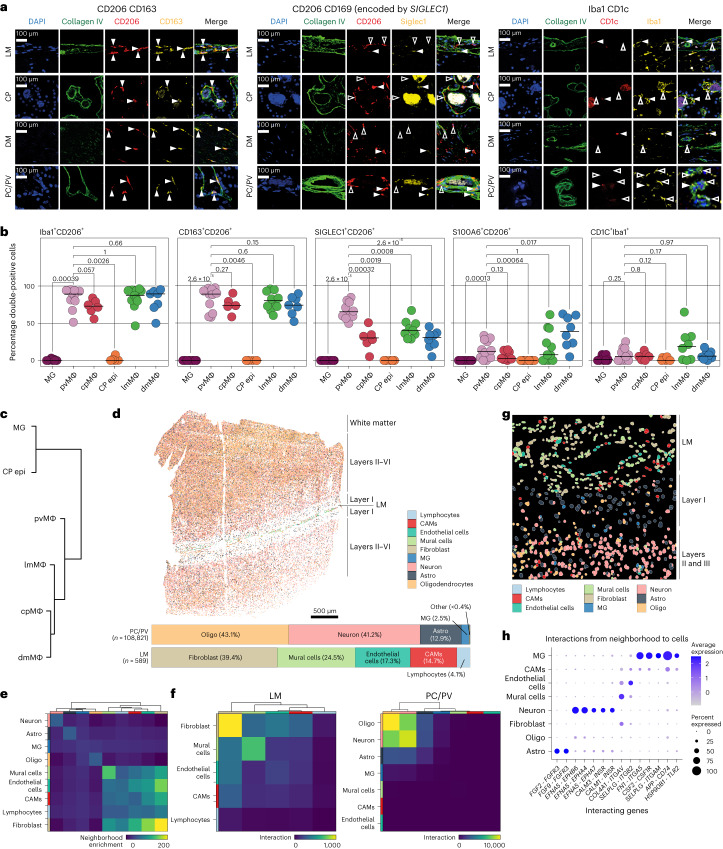
Spatial profiling of human CAMs in situ and their cellular interactions. **a**, Representative immunofluorescence across human CNS interfaces. DAPI (4′,6-diamidino-2-phenylindole) and collagen IV show positions of nuclei and basal lamina. Filled arrowheads indicate double-positive cells and empty arrowheads indicate single-positive cells. **b**, In situ quantification of selected markers across different compartments. Crossbars indicate medians. Outlier values confirmed by the Grubbs’s test were removed, resulting in at least *n* = 7 and at most *n* = 18 biologically independent samples analyzed per compartment and reaction. Each dot represents a patient. Indicated *P* values were derived from pairwise two-sided Mann–Whitney *U*-tests with pvMΦ as the reference cell type. MG, microglia; pvMΦ, perivascular macrophage; cpMΦ, choroir plexus macrophage; CP epi, epiplexus macrophage/Kolmer cells; lmMΦ, leptomengeal macrophage; dmMΦ, dura mater macrophage. **c**, Dendrogram showing the hierarchical clustering of the analyzed cell types based on average expression of CD206, SIGLEC1, CD163, S100A6 and CD1C. **d**, Spatial plot of a control section (frontal cortex) analyzed using ISS. Color coding represents different cell types. On the right, the different anatomical regions are annotated. The bar plot on the bottom shows the cell-type distribution in the PC and LM. Representative of cortical sections analyzed from four individuals. Astro, astrocytes; Oligo, oligodendrocytes. **e**, Heat map, color coded for the neighborhood enrichment scores of the different cell types calculated with a permutation-based test^36^. The color coding of the cell types is consistent with **d**. **f**, Heat map, color coded for the cell-type interaction scores in LM (left) and PC/PV (right). Color coding is consistent with **d**. **g**, Spatial plot of a tissue section (occipital cortex) analyzed using Nanostring CosMx. Color coding is consistent with **d** and **e**. Representative of 14 analyzed fields of view from 4 control samples. **h**, Dot plot showing the spatial cell–cell interactions of the dataset from **f**. The *y* axis indicates receiving cell types. The *x* axis labels show the ligand expressed by neighboring cells followed by the receptor. Color scale represents the average expression of ligand–receptor pairs. The dot size represents the percentage of cells expressing the receptor. Source data

**Extended Data Fig. 5:**
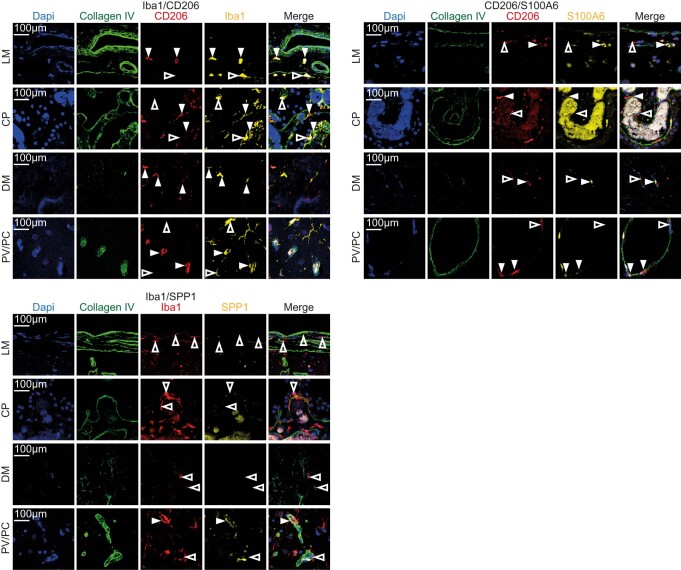
In-situ validation of immunohistochemistry markers identified in the scRNA-Seq data. Filled arrowheads indicate double-positive cells, empty arrowheads indicate single-positive cells. Scale bars correspond to 100 µm. At least 3 images per patient were analyzed.

We used collagen IV as a marker for basal lamina to confidently assign anatomical locations^9,10,12,24^. CD206 and CD163 were homogeneously coexpressed with Iba1 by CAMs across anatomical compartments, but not by microglia and Kolmer cells (Fig. 3a,b and Extended Data Fig. 5). Notably, CD169 was only expressed in up to two-thirds of CD206^+^ cells with the highest expression in perivascular CAMs. S100A6 and CD1C were very lowly expressed in the analyzed compartments. Hierarchical clustering based on average expression of the above-mentioned proteins confirmed the phenotypic similarity of microglia and Kolmer cells (Fig. 3c). Thus, we found enhanced expression of CD169 in PV CAMs.

For spatial transcriptome analysis, we applied ISS, a technique for optical detection of genes at a single-cell resolution^35^. The gene panel included cell-type markers and genes identified above with scRNA-seq (Supplementary Table 10). We analyzed a cortex specimen containing two cortical cross-sections separated by an LM layer (Fig. 3d). Azimuth-based cell-type annotation revealed distinct cellular compositions between the cortex and LM (Fig. 3d). Spatial neighborhood enrichment analysis^36^ showed that LM CAMs preferentially co-occurred and interacted with fibroblasts and endothelial cells (Fig. 3e,f). For cell–cell interaction analysis of human PC/PV and LM specimens, we used the Nanostring CosMx technology with a 1,000-plex gene panel (Supplementary Table 11). We observed an overlap between transcriptionally similar CAMs and microglia, with CD74 being the main receptor for CAM-directed neighborhood-to-cell interactions (Fig. 3g,h). In summary, spatial transcriptomics highlighted spatial interactions of CAMs.

### Microenvironment shapes developing human CAM phenotypes

**Fig. 4:**
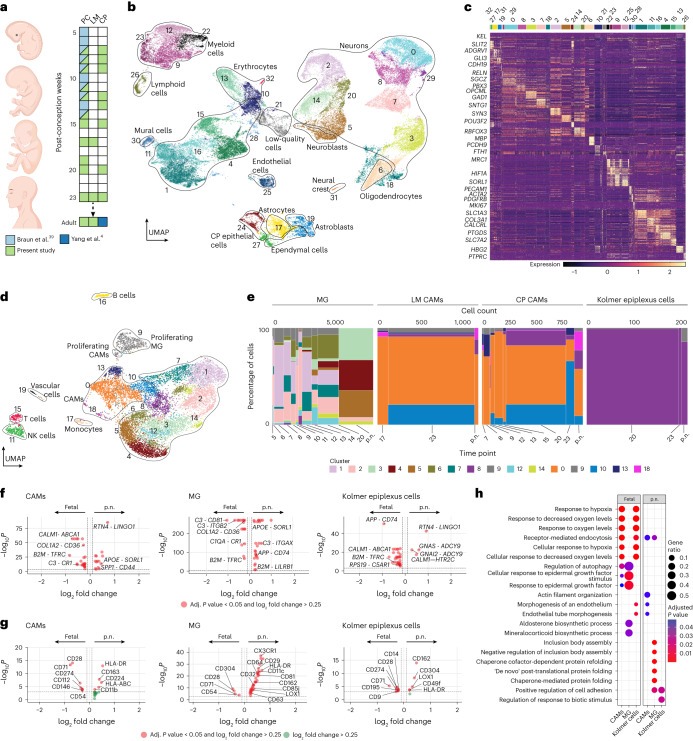
Region-dependent transcriptional dynamics of human CNS border macrophages during development. **a**, Schematic overview of the included time points and compartments color coded for the studies from which immune-cell data were integrated with the present data. **b**, UMAP visualization of single-nucleus RNA-seq data from 59,053 single-nucleus transcriptomes generated for the present study, color coded for Seurat v.4 clusters. **c**, Single-cell heat map depicting the gene expression of the top 20 marker genes per cluster of the cells shown in **b**. Selected genes are shown on the left-hand side. Color coding of the clusters is consistent with **b**. The color scale represents Pearson’s residuals from a regularized negative-binomial regression. **d**, UMAP visualization of single-nucleus RNA-seq data for immune cells generated and integrated immune-cell data from published studies^2,39^. The colors indicate the results of graph-based re-clustering using Seurat v.4. **e**, Marimekko charts showing the cluster contributions of the macrophage populations from **d** to the respective time points. Each macrophage population is plotted separately. p.n., postnatal. The color coding is consistent with **d**. **f**, Volcano plots showing differential gene expression testing of ligand–receptor pairs of the indicated macrophage populations from **d**. Selected representative ligand–receptor pairs are highlighted. A two-sided unpaired Wilcoxon rank-sum test was performed with Bonferroni correction for multiple testing. The color scale represents an adjusted *P* value below 0.05 and a log_2_ fold change above 0.25. **g**, Volcano plots showing differential surface protein expression derived using CITE-seq from the indicated fetal macrophages at pcw 23 with their postnatal counterparts. A two-sided unpaired Wilcoxon rank-sum test was performed with Bonferroni correction for multiple testing. The color coding represents an adjusted *P* value below 0.05 and a log_2_ fold change above 0.25. **h**, Gene Ontology enrichment analysis between fetal and postnatal macrophages from **g**. Gene Ontology testing was based on the top 100 differentially expressed gene per cell type and time point. Dot sizes indicate the ratio of marker genes per cluster over the genes of a given terms. The color scales encodes the Benjamini–Hochberg-adjusted *P* value from a one-sided Fisher’s exact test. Source data

**Extended Data Fig. 6:**
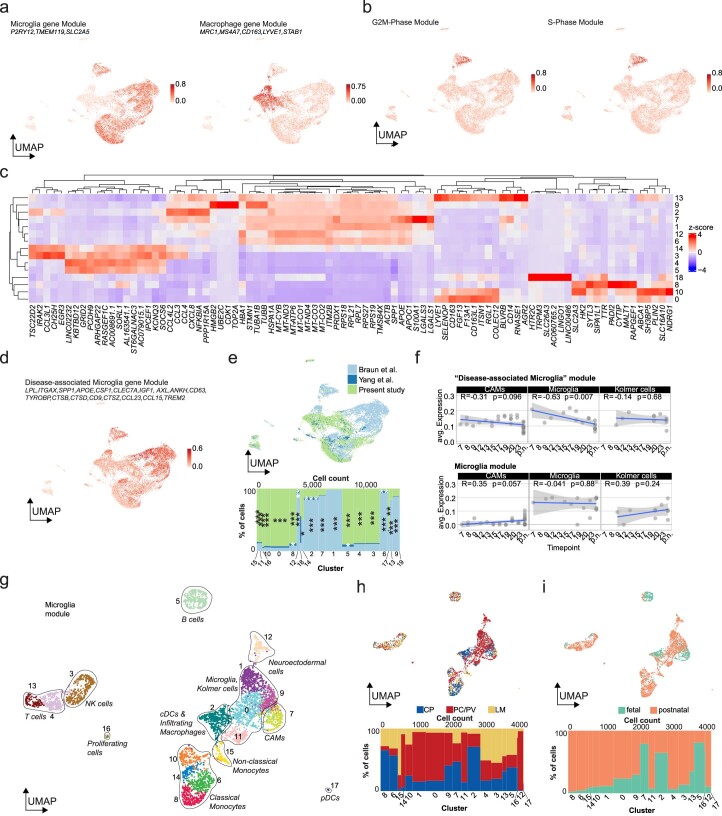
Single-nucleus and single-cell profiling of fetal and postnatal tissues. **a**. UMAPs color coded for the expression of cell type-enriched gene modules. The color coding represents gene module scores using the Mann-Whitney U statistic. **b**. UMAPs color-coded for cell cycle scoring calculated using the Mann-Whitney U statistic. The underlying gene modules were previously described^27^. **c**. Heatmap of the average expression of the 7 top markers in the indicated cell types. The color scale indicates the z-score. The dendrogram represents the hierarchical clustering based on Euclidean distances. **d**. UMAP color-coded for the expression of the published antigen-presentation and DAM modules^7,29^. The color coding represents gene module scores using the Mann-Whitney U statistic. **e**. UMAP (top) and Marimekko chart (bottom) color-coded for the dataset of each cell. The Marimekko chart represents the contribution of each dataset to the respective cluster. Asterisks indicate statistical significance from one-sided hypergeometric tests with Benjamini–Hochberg adjustment for multiple testing. **p < 0.01; ***p < 0.001. Note that for enhanced readability, asterisk are only indicated up to C17. **f**. Dot-line plots showing the average expression of the macrophage (top), microglia (middle) and DAM modules per cell type across the developmental stages. To avoid pseudo-replication, gene expression was averaged for each cell type and patient. Each dot represents one donor. The lines indicate linear regression results with the confidence intervals displayed as shaded areas. Pearson correlation coefficient and p value are given at the top of each plot. **g**. UMAP of 4,332 FACS-sorted CD45+ cells from the PC/PV, LM and CP of a fetus from pcw 23 and adult controls. The color coding indicates Seurat clusters. The cell-type assignment is based on published datasets^2,38^. **h**. UMAP (top) and Marimekko chart (bottom) color-coded for the anatomical compartment each cell was derived from. The Marimekko chart represents the contribution of each compartment to the respective cluster. The postnatal samples are from 5 individuals with 2 samples each from the PC/PV, CP and LM. **i**. Equivalent of figure panel h color-coded for developmental timepoints. Source data

Myeloid cells enter the human CNS by post-conception week (pcw) 4.5 (ref. ^37^). Although fetal human microglia have previously been profiled^38,39^, comprehensive single-cell analyses of developmental human CAMs are unavailable. Hence, we conducted single-nucleus RNA-seq on 59,053 cells from 32 frozen human PC/PV, LM and CP samples between pcw 7 to 23 and postnatal PC/PV and LM specimens. Additionally, we integrated our immune-cell transcriptomes with published data (Fig. 4a). We FACS-sorted NeuN^−^Olig2^−^ nuclei and obtained 59,053 nuclei from major neuroectodermal, structural and immune-cell types (Fig. 4b,c and Supplementary Table 12). For comparative analysis, the immune cells were integrated with published transcriptomes from human first-trimester PC/PV and adult control CP^2,39^. This approach yielded an anatomically dissected dataset of 13,807 immune-cell transcriptomes spanning the period of CNS myeloid cell engraftment through adulthood. We mainly found myeloid cells with a comparatively large proportion of proliferating cells (Fig. 4d and Extended Data Fig. 6a,b). Notably, the proliferating C9 contained two subpopulations expressing either microglia or CAM genes (Extended Data Fig. 6a). Tracking cluster composition chronologically revealed microglia and CAM developmental dynamics. Before pcw 10, microglia were mainly C1 and C2 (Fig. 4e). C3, C4 and C5 became the majority after birth. For LM and CP CAMs, both before and after birth, the main clusters were C0 and C10. Kolmer cells (C8) were not observed before pcw 20, potentially due to under-sampling. Differential gene expression analysis highlighted *LYVE1*, *CD163* and *F13A1* in CAMs (C0, C10 and C13) (Extended Data Fig. 6c). Kolmer cells (C8) mainly expressed *PADI2* and *SORL1*, also found in adult microglia (C4 and C5). Notably, first-trimester microglia (C1, C2 and C7) expressed chemokines and the DAM genes *SPP1* and *APOE*. Similar DAM gene module expression was previously observed^39^ (Extended Data Fig. 6d,e). Notably, even when excluding the Braun et al. data, a statistically significant time-dependent reduction in the DAM signature within microglia was evident, unlike in CAMs or Kolmer cells (Extended Data Fig. 6f). In summary, CAMs and microglia exhibited distinct transcriptional profiles early on, with the so-called DAM microglia signature inversely correlating with brain maturation.

We utilized cell–cell interaction analysis to uncover molecular mediators shaping CNS myeloid cell phenotypes. We merged the integrated immune-cell dataset with the non-immune-cell types from Fig. 4b and ran NICHES^40^ to infer interactions toward CAMs, microglia and Kolmer cells, respectively. To avoid sampling issues, we binned prenatal time points and compared them to the postnatal ones. Differential analysis identified transferrin receptor (*TFRC*) among the top prenatal receptors in all three cell types (Fig. 4f). CITE-seq analysis confirmed prenatal CD71 (encoded by *TFRC*) upregulation in these cells (Fig. 4g and Extended Data Fig. 6g–i). Postnatal cells upregulated immune mediators, including HLA-DR. Gene Ontology analysis identified hypoxia-related terms in prenatal CAMs and Kolmer cells and autophagy in CAMs and microglia (Fig. 4h and Supplementary Table 13). These results corroborate the importance of *TFRC* in hypoxia^41^. Postnatal CAMs were involved in vascular biological processes. In conclusion, we highlight the fetal brain milieu influencing gene and protein expression in brain myeloid cells with CAMs, microglia and Kolmer cells displaying a response to physiological prenatal hypoxia.

### Compartment-dependent turnover rates of CNS myeloid cells

**Fig. 5:**
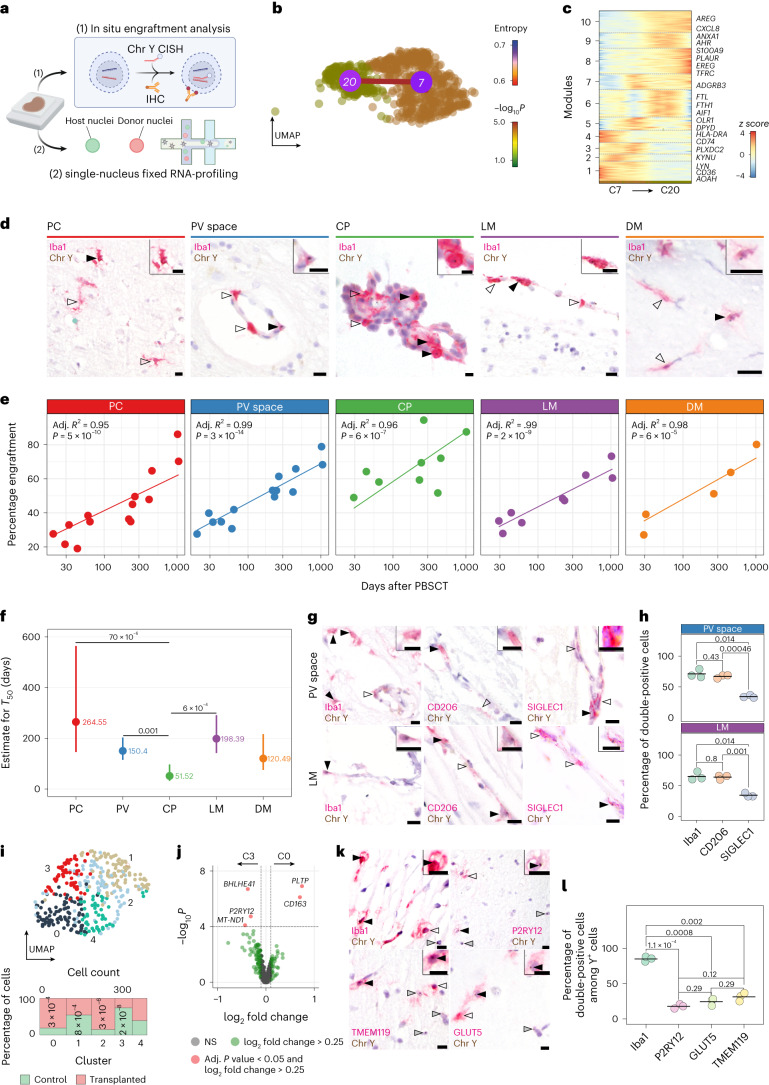
Compartment-specific turnover of human CNS border macrophages. **a**, Schematic workflow representation. IHC, immunohistochemistry; CISH, chromogen in situ hybridization. **b**, UMAP of C7 and C20 from Fig. 1b connected by a transcriptional trajectory. The color coding represents the log_10_ transformed *P* value indicating the overrepresentation of the trajectory compared to randomization^43^. The color of the cluster symbol encodes transcriptional entropy. **c**, Heat map showing stepwise gene expression along the trajectory. The color scale encodes *z* scores. **d**, Representative Iba1 immunohistochemistry and Y chromosome CISH. Filled arrowheads mark double-positive cells and empty arrowheads indicate single-positive cells. *n* ≥ 50 CAMs were analyzed per patient. Scale bars, 10 µm. **e**, Correlation between time after PBSCT and percentage of engraftment for *n* = 5 to *n* = 18 independent samples per compartment and one patient per dot. Adjusted *R*^2^ coefficients and one-sided *t*-test *P* values are given. **f**, Dot-whisker plots of the duration after PBSCT until 50% engraftment for the data from **e**. Whiskers indicate 95% confidence intervals around the predicted mean value. *P* values were calculated from pairwise comparisons using estimated marginal means with Tukey’s multiple testing adjustment. **g**, Representative immunohistochemistry of PV and LM. Filled arrowheads indicate double-positive cells and empty arrowheads indicate single-positive cells. Scale bars, 10 µm. *n* ≥ 3 fields of view per patient were quantified. **h**, Quantification of the dataset in **g**. *n* = 3 biologically independent samples were analyzed per compartment. *P* values indicate pairwise two-sided *t*-tests with Holm–Bonferroni multiple testing adjustment. **i**, UMAP of single-nucleus fixed mRNA profiling of *n* = 363 myeloid cells color coded for clusters. The Marimekko chart (bottom) indicates the distribution of conditions per cluster. Significant one-sided hypergeometric test *P* values with Benjamini–Hochberg multiple-testing adjustment are shown. **j**, Volcano plot showing differentially expressed genes between the control-enriched C3 and PBSCT-enriched C0. Two-sided unpaired Wilcoxon rank-sum tests with Bonferroni multiple testing adjustment were performed. The color coding is explained below the graph. NS, not significant. **k**, Representative immunohistochemistry in the PC. Filled arrowheads indicate double-positive cells and empty arrowheads indicate single-positive cells. Gray arrowheads indicate single-positive Y^+^ cells. *n* ≥ 3 fields of view per patient were quantified. Scale bars, 10 µm. **l**, Quantification of the dataset in **k**. *n* = 3 independent samples were analyzed per compartment and one patient per dot. *P* values were calculated using pairwise two-sided *t*-tests with Holm–Bonferroni multiple-testing adjustment. Source data

Turnover of CAMs by circulating cells has been investigated in mice^9,10,24^ but not in humans. Hence, we established in situ fate mapping in human brain tissues from 15 sex-mismatched peripheral blood stem cell transplantation (PBSCT) recipients^42^. This analysis involved quantifying Y chromosome-positive (Y^+^) immune cells and genome-wide single-nucleus transcriptome profiling (Fig. 5a). Pseudotime analysis^43^ of intravascular and homing monocytes (C7 and 20 from Fig. 1b) revealed sequential transcriptional changes during myeloid cell engraftment (Fig. 5b). This was marked by an upregulation of chemokine and myeloid homing (*CXCL8*, *AREG* and *PLAUR*)^29^ and iron-scavenging genes (*TFRC*, *FTL* and *FTH1*) (Fig. 5c and Supplementary Table 14).

Next, we quantified bone-marrow-derived engrafted cells at the CNS interfaces of female patients who received sex-mismatched PBSCT to treat hematological diseases (Supplementary Table 20). Notably, we observed donor-derived Y^+^Iba1^+^ cells within all examined CNS interfaces and the brain PC (Fig. 5d). Correlation analysis between the percentage of Y^+^Iba1^+^ cells and all Iba1^+^ cells in PC/PV, CP, LM and DM clearly demonstrated a time-dependent increase of Y^+^Iba1^+^ cells (Fig. 5e). Of note, *T*_50_, the time to reach a 50% exchange rate of Y^+^Iba1^+^ cells, displayed significant variability across compartments ranging from 51.52 d (95% confidence interval 31.9–97.1) in the CP to 264.55 d (95% confidence interval 145.9–563.9) for the brain PC (Fig. 5f). Notably, the *T*_50_ exhibited substantial dispersion, particularly within the parenchyma, suggesting influence from interindividual characteristics. Given CD206 expression in nearly all CAMs whereas SIGLEC1 was only present in a subset (Fig. 3b), we wondered whether Y^+^CD206^+^ and Y^+^SIGLEC1^+^ cells were found at different rates. Indeed, at late post-transplantation time points (218, 453 and 1,018 d) Y^+^SIGLEC1^+^ cells were present in approximately half of the Y^+^Iba1^+^ and Y^+^CD206^+^ cells (Fig. 5g,h).

**Extended Data Fig. 7:**
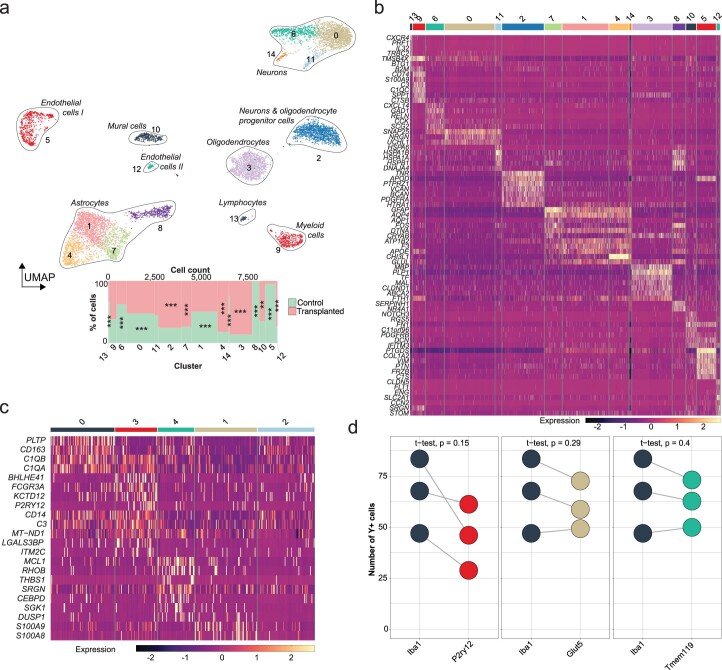
Profiling of engrafting myeloid cells. **a**. UMAP visualization of 9,035 color-coded for Seurat v4 clustering results. Cell type assignment was conducted based on published gene expression signatures. Marimekko chart of the contribution of control and transplanted patient to each cluster. Asterisks indicate the results of statistical testing using one-sided hypergeometric tests. Adjustment for multiple testing was done using the Benjamini–Hochberg method. **p < 0.01; ***p < 0.001. **b**. Single-cell heatmap depicting the gene expression of the top 7 cluster marker genes per cluster of the cells shown in panel a. The genes are shown at the left-hand side of the heatmap. Color-coding of the cluster is consistent with panel a. The color scale represents Pearson’s residuals from a regularized negative-binomial regression. **c**. Single-cell heatmap depicting the gene expression of up to top 10 cluster marker genes per cluster of the myeloid cell cluster from Fig. 4 i. **d**. Comparative analysis of the percentage of Y+ positive cells among all identified Iba1 + , P2RY12 + , TMEM119+ and GLUT5+ cells in the cortex. Positivity for the pan-myeloid marker Iba1 was considered as the maximum achievable percentage of engrafting myeloid cells. The lines connect the dots between results from the same patients. The indicates p values were calculated using paired t-tests. Source data

For a deeper profiling of engrafted cells, we conducted single-nucleus mRNA profiling from formalin-fixed paraffin-embedded (FFPE) tissues of the above-mentioned three post-transplantation samples and age-matched controls. We FACS-sorted NeuN^−^Olig2^−^ nuclei from PC/PV and adjacent LM sections, yielding 9,035 single-nucleus transcriptomes of the major brain, vascular and immune-cell types (Extended Data Fig. 7a,b). Subclustering 363 myeloid cells identified two adjacent clusters (C3 and C0) enriched in control and transplanted patients, respectively (Fig. 5i). Both clusters included *C1QA* and *C1QB* among top markers, indicative of microglia and tissue-resident macrophages (Extended Data Fig. 7c and Supplementary Table 15). The control-enriched C3 expressed the microglia-enriched genes *BHLHE41* and *P2RY12*, whereas the post-transplantation-enriched C0 showed upregulation of *CD163* and *PLTP*, implying an activated phenotype (Fig. 5j).

Given the potential comorbidities in PBSCT patients, we analyzed the immune phenotype of resident microglia and CAMs using in situ fate mapping analysis of PC cells (Fig. 5k). Notably, 90% of Y^+^ cells were double-positive for Iba1, but only about 20% of Y^+^ cells expressed microglia core markers P2RY12, GLUT5 and TMEM119 (Fig. 5l). When analyzing the percentage of Y^+^ cells among all Iba1^+^, P2RY12^+^, TMEM119^+^ and GLUT5^+^ cells in the same three cases, we found no major differences between brain-resident and engrafting cells (Extended Data Fig. 7d). In summary, we described patterns of human brain myeloid-derived cell engraftment and evidenced compartment-specific engraftment rates across different CNS interfaces. High-dimensional profiling was constrained by the scarcity of these regions, we revealed that cells engrafting in the parenchyma maintained an activated phenotype.

### Context-dependent CAM signatures in human patients with glioblastoma

**Fig. 6:**
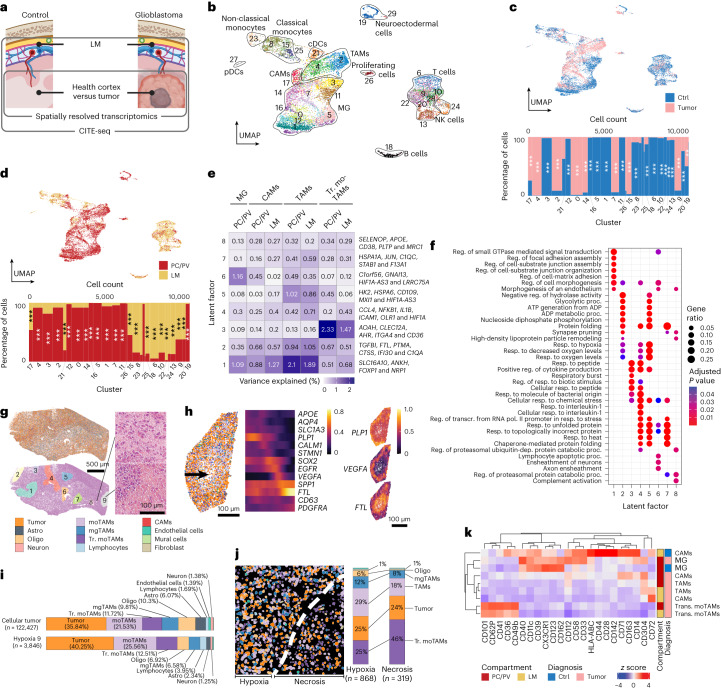
Multimodal analysis reveals common activation programs of CAMs and microglia in human glioblastoma. **a**, Schematic workflow representation. **b**, UMAP of *n* = 11,681 FACS-sorted cells color coded for clusters. CP and DM samples were not available from glioblastoma. **c**, UMAP (top) and Marimekko chart (bottom) color coded for the underlying diagnosis. The Marimekko chart represents the distribution of diagnoses per cluster. One-sided hypergeometric tests with Benjamini–Hochberg multiple testing adjustment were performed. *P < 0.05; **P < 0.01; ***P < 0.001. Exact *P* values are found in Supplementary Table 16. For improved readability, significance asterisks are shown until C24. C25–C29 represent relatively small cell numbers. **d**, Equivalent of **c** analyzed for anatomical compartments. Exact *P* values are found in Supplementary Table 18. **e**, Heat map of MOFA2 latent factors of glioblastoma-associated macrophage populations present in both compartments. The color-coded data and the values of variance explained are indicated in each heat map tile. Representative genes are presented per factor. **f**, Gene Ontology analysis of the top 100 marker genes per MOFA2 factor. Dot sizes indicate the gene ratio per cluster. Color coding of the dot encodes the Benjamini–Hochberg-adjusted *P* value based on a one-sided Fisher’s exact test. Reg., regulation; Resp., response. **g**, Spatial plot of a glioblastoma section analyzed with ISS and color coded for cell types (top) and histological subtypes (left). The numbered areas represent hypoxic regions. Samples from four patients were analyzed. Representative hematoxylin and eosin staining is shown on the right. **h**, Spatial plot color coded for the cellular composition of hypoxic area 9 from **g**. The arrow represents a spatial trajectory from the periphery to the hypoxic core. The heat map (center) shows stepwise gene expression along the trajectory with representative genes (right). **i**, Bar plots of the cell-type compositions of cellular tumor and hypoxic regions representative of sections analyzed from two individuals. **j**, Spatial plot of the transition from hypoxia to necrosis analyzed with Nanostring CosMx. Color coding is specified in **h**. The bar plots show the cell-type distribution. The data are representative of eight regions from two glioblastoma samples. **k**, Mean marker expression heat map of CITE-seq data from microglia, CAMs and monocyte-derived macrophages shown in **b**. The compartments and diagnoses of the cells are color coded. Source data

**Extended Data Fig. 8:**
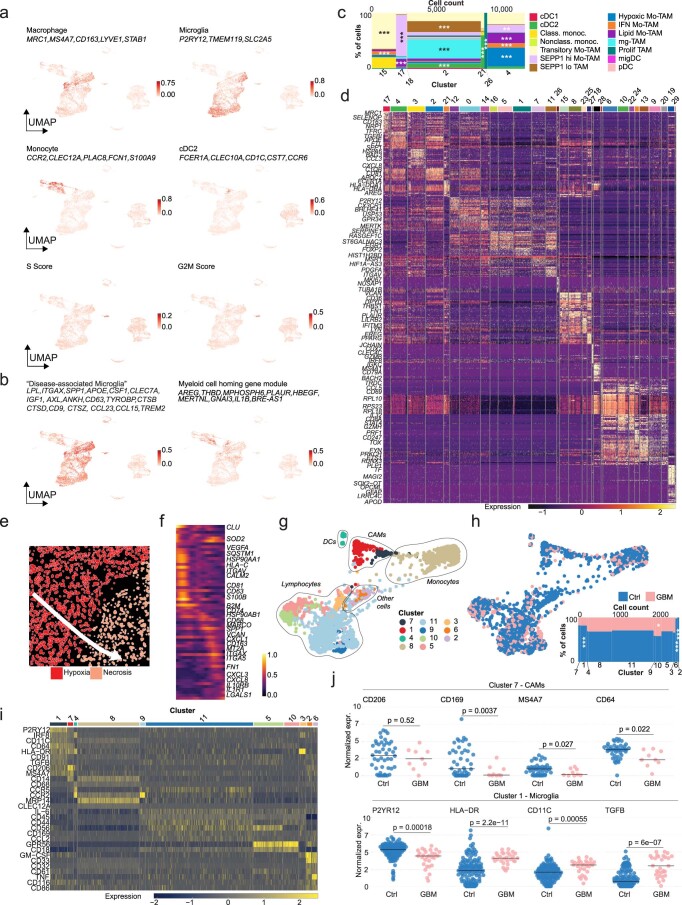
Validation of control vs GBM-associated immune cells assessed by single-cell RNA-Seq and mass cytometry. **a**. UMAP color-coded for the expression of the indicated modules scores. Module scores are based on the Mann-Whitney U statistic. The cell cycle module genes are published^27^. **b**. UMAP color-coded for DAM and myeloid cell homing module expression^28,29^. Module scores are based on the Mann-Whitney U statistic. **c**. Marimekko chart showing the integration results of the present dataset with published data^43^. Cluster assignments of the present data were transferred on the Antunes et al dataset. Asterisks indicate statistical significance from one-sided hypergeometric tests with Benjamini–Hochberg adjustment for multiple testing. *p < 0.05; **p < 0.01; ***p < 0.001. **d**. Single-cell heatmap showing the expression of the top 20 cluster markers with selected genes shown on the left. Cluster colors are consistent with panel b. The color scale represents Pearson’s residuals from a regularized negative-binomial regression. **e**. Spatial plot of hypoxia (red) and adjacent necrosis (orange) analyzed with Nanostring CosMX. The white arrow indicates a spatial trajectory shown in panel f. The shown data is representative of 8 analyzed fields from 2 glioblastoma samples. **f**. Spatial trajectory heatmap showing the smoothed gene expression along the trajectory from figure panel e with representative genes on the right. **g**. Spider visualization of mass cytometry data from 2,439 CD45^+^ glioblastoma-derived cells color-coded for clusters. Cell type assignment is based on published cell type marker expression. CD45^-^ cells are labeled as ‘others’. **h**. Spider presentation (top) and Marimekko chart (bottom) showing the distribution of diagnoses per cluster. **i**. Protein expression single-cell heatmap of the cells in panel e. The color scale represents Pearson’s residuals from a regularized negative-binomial regression. **j**. Dot-line plot depicting the expression of selected proteins between tumor-associated and control CAMs in C7 from panel e (top) and microglia from C1 (bottom). The indicated p values were calculated using unpaired two-sided t-tests with Benjamini–Hochberg multiple testing adjustment. Each symbol represents one cell. Medians are indicated. Source data

Glioblastoma are malignant brain tumors with a complex immune microenvironment^33,44^. We profiled 11,681 CAMs from 21 anatomically dissected glioblastoma and control samples using scRNA-seq, CITE-seq and spatial transcriptomics (Fig. 6a). Alongside normal brain cell types, tumor samples contained tumor-associated macrophages (TAMs)^33,44^ in C2 and C4 (Fig. 6b,c and Supplementary Table 16). C2 cells expressed microglia genes, whereas C4 showed macrophage gene expression, corresponding to microglia TAMs (mgTAMs) and monocyte-derived TAMs (moTAMs), respectively (Extended Data Fig. 8a). Additionally, the tumor-enriched C15 contained homing monocytes resembling transitory moTAMs (Tr. moTAMs)^44^ (Extended Data Fig. 8b and Supplementary Table 16). Label transfer of our clusters onto published data^44^ confirmed Tr. moTAMs in C15, mgTAMs in C2 and hypoxic and lipid moTAMs in C4 (Extended Data Fig. 8c). Notably, so-called SEPP1^hi^ moTAMs were indistinguishable from control CAMs in C17, mirroring their high *SELENOP* expression (previously known as *SEPP1*)^44^ (Fig. 1d, Extended Data Fig. 8d and Supplementary Table 17).

Cluster analysis assessed PC/PV and LM contributions to each cluster. Besides intravascular lymphoid cells, LM contained cDC (C21) and Tr. moTAM (C15) (Fig. 6d and Supplementary Table 18). TAMs (C2 and C4) were enriched in PC/PV, suggesting some CAM involvement in the glioblastoma microenvironment.

To dissect distinct transcriptional variations within the glioblastoma immune microenvironment, we focused on tumor-associated myeloid cells and employed MOFA2. Among TAMs, latent factors 1, 2 and 5 were expressed (marker genes *NRP1*, *FTL* and *HIF1A-AS3*) (Fig. 6e and Supplementary Table 19). Notably, PC/PV and LM CAMs were primarily characterized by latent factor 1, whereas latent factor 3 (marker genes *CLEC12A* and *CD36*) explained the variability in Tr. moTAMs. Microglia variability was mainly explained by latent factor 6 (marker gene *HIF1A-AS3*). Notably, latent factor 4 (marker genes CCL4, IL1B and ICAM1) was found in LM-derived cells, highlighting the role of the LM as gateway for engraftment. Gene Ontology analysis of latent factors yielded terms associated with the cell matrix and vasculature for latent factor 1, hypoxia for factors 2, 4 and 5, and immune activation for factors 3 and 4 (Fig. 6f). Considering the influence of hypoxia on TAM transcriptional profiles, we analyzed their distribution within the tumor. ISS on a tumor section spanning several mm^2^ identified nine hypoxic regions with focal *VEGFA* expression amid cellular tumor regions (Fig. 6g). Spatial trajectory analysis from the periphery to the hypoxic center of area nine unveiled decreasing *PLP1* and *EGFR* expression, followed by a focal peak of *VEGFA* at the rim and *FTL* expression at the core (Fig. 6h). Corresponding with *FTL* expression in MOFA2 latent factor 2, more moTAM were in hypoxic area 9 than the surrounding tumor (Fig. 6i).

To further dissect the perinecrotic area, we employed Nanostring CosMx technology. This revealed a significant rise of moTAMs and Tr. moTAMs toward the necrosis (Fig. 6j). Mirroring ISS, spatial trajectory analysis demonstrated the downregulation of neuroectodermal genes and the upregulation of myeloid genes at the perinecrotic area (Extended Data Fig. 8e,f).

To identify protein markers, we conducted differential expression analysis of the CITE-seq data. Tumor-associated microglia downregulated CX3CR1 but upregulated CD112, CD58 and CD33 (Fig. 6k). Tumor-associated CAMs downregulated CD163 and upregulated CD304 (encoded by *NRP1*). *NRP1* as one of the markers of MOFA2 latent factor 1, was also upregulated in TAMs and microglia, but not Tr. moTAMs. Mass cytometry with additional markers, demonstrated unchanged levels of CD206 but reduced CD169, MS4A7 and CD64 in glioblastoma-associated CAMs (Extended Data Fig. 8g–j). Glioblastoma-associated microglia reduced P2Y12 and increased HLA-DR, CD11c and TGFβ expression.

In summary, glioblastoma contained varied myeloid populations, abundant in hypoxic and necrotic tumor areas. Within microglia, CAMs and TAMs we observed CD304 (encoded by *NRP1*) upregulation, a feature absent in Tr. moTAMs. LM TAMs displayed enhanced chemokine and integrin expression, potentially linked to LM’s role as gateway to the tumor. Our comprehensive spatial profile deepens the understanding of the glioblastoma-associated myeloid compartment.

## Discussion

**Extended Data Fig. 9:**
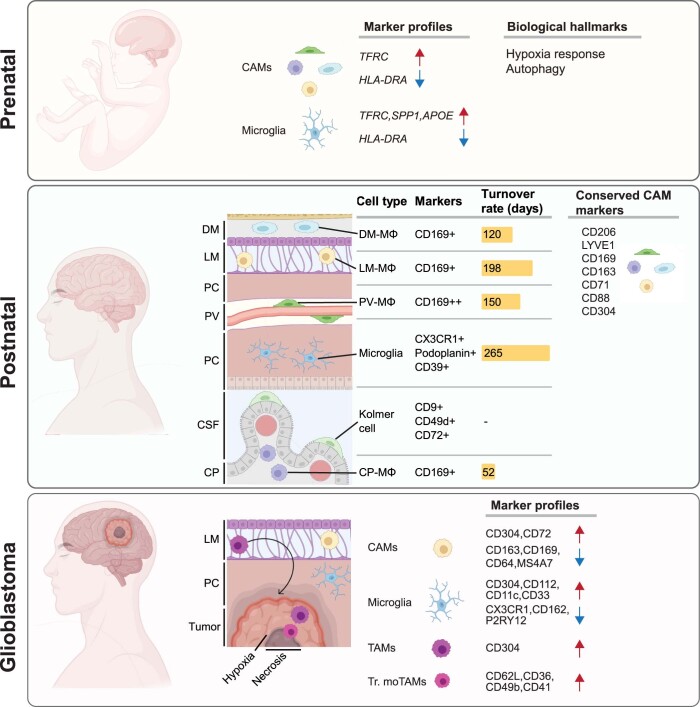
Graphical abstract of the present study. The identified markers were chosen based on the combined findings of the modalities.

This study provides a comprehensive exploration and functional analysis of human CNS interfaces during development, homeostasis and glioblastoma (Extended Data Fig. 9). Different scRNA-seq protocols, CITE-seq, mass cytometry and high-resolution spatial transcriptomics unveiled diverse immune-cell interactions within human PC/PV, LM, CP and DM. CAMs consistently exhibit a core macrophage signature (*MRC1*, *LYVE1* and *F13A1*). CP contains microglia-like Kolmer cells, whereas transitory monocytes predominate in CP and DM, sustaining myeloid-derived engraftment. Histological validation confirms varied CD169 (encoded by *SIGLEC1*) expression across compartments. Cross-species comparison underscored evolutionary CAM marker conservation in humans and mice. High-resolution spatial transcriptomics implied CAMs crosstalk within their niche. Investigation of in vivo engraftment dynamics at CNS interfaces revealed varying engraftment rates across compartments.

Comparing fetal and adult CNS myeloid cells uncovered distinct populations. Fetal CAMs and microglia were already transcriptionally distinct at pcw 5. Fetal CAMs, microglia and Kolmer cells expressed attenuated immune-mediator proteins and heightened hypoxia markers (CD71). Likewise, glioblastoma-associated LM and PC/PV CAMs exhibited hypoxia-responsive traits, with *FTL* and *NRP1* (encoding CD304) upregulation. High-resolution spatial analysis highlighted a differential distribution of myeloid population across glioblastoma regions.

In summary, our study significantly advances our understanding of human CNS myeloid cells: (1) providing comprehensive transcriptomic and proteomic profiles across human microglia and CAMs along with spatial profiling of the LM and PC/PV niches; (2) offering direct evidence of in vivo peripheral myeloid cells engraftment into human CNS parenchyma and interfaces with concurrent transcriptomic profiling of these cells; (3) highlighting distinct gene and surface protein expression patterns between fetal and postnatal CAMs; and (4) conducting a spatially resolved profiling of the PC/PV and LM glioblastoma microenvironments.

Unlike microglia^10,45^ few studies have explored immune cells at human CNS interfaces. Previous comprehensive transcriptomic profiling of CNS interfaces occurred in mice^7,8^. Limited CAM populations are reported in separate human and murine PV^4–6^ and DM^46^ datasets. We have recently shown that PV CAMs are postnatally derived from LM CAMs, implying functional similarity^24^. Functionally, PV CAMs have been linked to cerebrovascular scavenging and dysfunction^47–50^ supported by our findings of enhanced CD169 scavenging receptor expression in PV.

Distinct CAMs and microglia populations are present in first-trimester human brains. Fetal hypoxia seemed pivotal in shaping myeloid cell phenotypes. Notably, glioblastoma-associated CAMs and TAMs exhibited genes linked to metal iron homeostasis and angiogenesis. Disrupted iron metabolism is a hallmark of cancer-associated hypoxia and anticancer defense^3,41^. Previous research has shown fetal-like progenitor cell states in glioblastoma^51^. We and others reported hypoxia-responsive states in glioblastoma-associated^32,33,52^ and multiple sclerosis-associated^53^ microglia, highlighting a potentially generic reactive myeloid cell state.

Assessing in vivo engraftment of bone-marrow-derived cells into the adult human CNS is challenging. While microglial engraftment is established in mice^42,54,55^, insights into human CNS interface engraftment is lacking. We quantified Y^+^Iba1^+^ donor-derived MΦ in female PBSCT autopsy cases showing varying time-dependent engraftment rates. Transcriptional analysis indicated sustained activation in engrafted cells. Our findings demonstrate human CNS interface myeloid cell engraftment, suggesting PBSCT as a potential CNS-wide myeloid cell replacement strategy for disorders associated with myeloid cell abnormalities^10^.

Despite several strengths, our study has limitations. The rarity of CAMs at CNS interfaces necessitated enrichment strategies for adequate cell numbers. Tissue scarcity was another limiting factor, particularly for DM, CP and fetal specimens. Transcriptional convergence of moMΦ and CAMs under pathological conditions renders these cell types difficult to distinguish^7^. We addressed these issues through a semi-supervised cell classification approach using published transcriptional signatures^27,32,44^; however, lineage-tracing in transgenic mice surpasses this approach^7,9,22^. Additionally, our study is limited by the inherent biases in scRNA-seq^56^.

To conclude, we comprehensively profiled human CNS myeloid cells, focusing on CAMs during homeostasis and disease. We detail the transcriptional spectra of human CAMs and moMΦ states, surface protein profiles, species comparison and perform various validation experiments. Furthermore, we uncover distinct transcriptional profiles of fetal CAMs and identify some similarities to glioblastoma-associated CAMs, driven by different pathophysiological contexts. Notably, myeloid cell engraftment at human CNS interfaces opens the doors to potential CAM replacement therapies for diseases rooted in CAM dysfunction.

## Methods

### Prospective tissue collection

Experiments on human tissue samples were performed in accordance with the Declaration of Helsinki. Tissues from different sources were analyzed in the present study. The analyzed anatomical compartments include LM (*n* = 55), PC/PV (*n* = 52), CP (*n* = 23), and DM (*n* = 8). Overall, 61 donors were female and 41 were male. An overview of the sample characteristics is provided in Supplementary Table 20. In short, single-cell and single-nucleus RNA-seq analyses of fresh or fresh-frozen postnatal samples were conducted under the oversight of the local Research Ethics Committee of the University Freiburg Medical Center under the protocol numbers 472/15 and 253/17. Patients or their legal guardians provided written informed consent before tissue collection. Analysis of fetal tissues from the University of Freiburg Medical Center and the Human Developmental Biology Resource (HDBR) were conducted under the oversight of local Research Ethics Committee of the University Freiburg Medical Center under the protocol number 253/17 and the National Research Ethics Service in the United Kingdom. The embryonal and fetal samples were collected under the supervision of specialists and lay persons. Tissue for the HDBR is donated voluntarily after providing informed consent from collaborating clinics in the United Kingdom. Analyses of the adult autopsy tissues were conducted under the oversight of local Research Ethics Committee of the University Freiburg Medical Center under the protocol numbers 10008/09 and 472/15 and the local committees associated with the National Institutes of Health (NIH) bio banks with written informed consent provided by the patients or their legal guardians.

Radiologically healthy or tumor tissues obtained at University of Freiburg Medical Center were placed in ice-cold PBS after surgical removal. Macroscopically, control tissues from focal epilepsy, primary and secondary brain neoplasm surgeries were selected based on radiological appearance and >2 cm distance from the lesion. Control cases that passed pathological examination were included in the study. To assess glioma-associated molecular changes, IDH-wild-type glioblastoma, CNS World Health Organization grade 4 were included (Supplementary Table 20). For single-nucleus RNA-seq, fresh-frozen biobanked tissues were used. No statistical methods were used to predetermine sample sizes, but our sample sizes are similar to those reported in previous publication^44,57,58^. The study was not designed to detect sex differences and no analyses were performed regarding this question. Sex was derived from records.

### Immunofluorescence

Data collection and analysis were performed blind to the conditions of the experiments. FFPE sections were blocked and permeabilized with PBS containing 5% normal donkey serum and 0.5% Triton-X 100 for 1 h at room temperature (RT). Primary antibodies were incubated overnight with combinations of Iba1 (WACO), Iba1 (Synaptic Systems), MRC1 (also known as CD206) (Abnova), CD1C (Abcam), CD163 (Sigma-Aldrich), S100A6 (Sigma-Aldrich), SIGLEC1 (also known as CD169) (Sigma-Aldrich) and collagen IV (Sigma-Aldrich,). Secondary antibodies were added as follows: Alexa Fluor 488 1:500 dilution (Thermo Fisher Scientific), Alexa Fluor 568 1:500 dilution (Thermo Fisher Scientific), Alexa Fluor 647 1:500 dilution (Thermo Fisher Scientific) and Alexa Fluor 647 (Jackson ImmunoResearch Laboratories) 1:500 dilution for 2 h at RT. Nuclei were counterstained with DAPI (Carl Roth) when necessary. Images were taken using conventional fluorescence microscopes (Olympus BX-61 and Keyence BZ-9000) and confocal pictures were taken with a Leica TCS SP8 (Leica). Image quantification was conducted in Adobe Photoshop. Outlier values were identified using Grubbs’s test in R and removed.

### Tissue dissection for single-cell suspensions and flow cytometry

Before flow cytometry, tissues were digested in Accumax (Sigma-Aldrich) for 30 min at RT with orbital shaking at 800 r.p.m. LM were removed from PC samples and digested separately. All subsequent processing steps were performed on ice. After digestion, tissue samples were suspended in ice-cold Hank’s balanced salt solution (Thermo Fisher Scientific) containing 10 mM glucose (Thermo Fisher Scientific) and 10 mM HEPES (Thermo Fisher Scientific) and mechanically dissociated using glass shearing with a 10-ml Potter–Elvehjem pestle and glass tube homogenizer (Merck). Larger tissue debris were removed by filtering through a 70-µm cell strainer (BD Bioscience). After centrifugation, cell pellets were cryopreserved in fetal calf serum:DMSO (9:1; Merck) until further processing. To minimize batch effects, experiments were conducted in a blocked manner with tissues from different regions and control and diseased tissues processed together using commercial multiplexing kits (10x Genomics, see below for details). Also, where possible several control tissues were processed on the same day. Single-cell sorting was performed on a MoFlo Astrios (Beckman Coulter). Anti-CD45 (clone HI30, APC, BD Bioscience) antibodies were used for droplet-based single-cell RNA-seq (Extended Data Fig. 1a). The following antibodies were used for Cel-Seq2 sorting: anti-CD45 (1:100 dilution, clone HI30, APC, BD Bioscience), anti-MRC1/CD206 (1:400 dilution, clone 15-2, APC-Cy7, BioLegend), anti-CD3 (1:100 dilution, clone SP34-2, PE-Cy7), anti-CD11b (1:800 dilution, clone M1/70, eBioscience), anti-CD19 (1:100 dilution, clone SJ25C1, PE-Cy7, BioLegend) and anti-CD20 (1:400 dilution, clone 2H7, PE-Cy7, BioLegend). Before surface staining, Fc receptors were blocked using Human TruStain FcX (BioLegend). DAPI staining was used for dead cell removal.

### Single-nucleus preparations from frozen tissues for flow cytometry

For single-nucleus RNA-seq from fresh-frozen tissues, we utilized an adaptation of the Frankenstein community protocol (www.protocols.io/view/frankenstein-protocol-for-nuclei-isolation-from-f-5jyl8nx98l2w/v3). All steps were performed at 4 °C. Briefly, a tissue piece the size of a grain of rice was mechanically dissociated in Nuclei EZ Lysis Buffer (Merck) using a pellet pestle (Merck). The resulting homogenate was incubated on ice and filtered with a 70-µm cell strainer (Merck). Subsequently, cells were centrifuged at 500*g* for 5 min and incubated for 5 min with Nuclei EZ Lysis Buffer, centrifuged again and incubated for 5 min in nuclei resuspension buffer (PBS supplemented with 1% BSA solution (Miltenyi Biotec) and 0.2 U μl^−1^ RNase inhibitor (New England Biolabs)). After two wash steps with nuclei resuspension buffer, nuclei were incubated for 20 min with a master mix containing DAPI (10 µg ml^−1^), anti-NeuN (clone 1B7, Alexa-647, Novus Biologicals) and anti-Olig2 (clone 211F1.1, Alexa-488, Merck). Single DAPI^+^NeuN^−^Olig2^−^ nuclei were sorted on a MoFlo Astrios (Beckman Coulter) or BD FACSAria III machines (BD Bioscience) to enrich for non-neuroectodermal cell types (Extended Data Fig. 1a). To minimize batch effects, experiments were conducted in a blocked manner with tissues from different regions and control and diseased tissues processed together using commercial multiplexing kits (10x Genomics, see below for details).

### Surface protein profiling

CITE-seq was conducted using a commercially available human antibody cocktail (TotalSeq-B Human Universal Cocktail, v.1.0, BioLegend). The lyophilized cocktail was dissolved in Cell Staining Buffer (BioLegend) following the manufacturer’s instructions. After Fc receptor blocking, cell suspensions were incubated with the dissolved antibody solution for 30 min and washed twice.

### Sample multiplexing using lipid-conjugated cell-multiplexing oligonucleotides

Sample multiplexing with the 3′ CellPlex kit (10x Genomics) was applied for cost efficiency to pool up to 12 samples per 10x reaction. Cell and nucleus suspensions were incubated on ice for 20 min followed by three washes.

### Fixed RNA profiling

Nuclei were extracted from FFPE tissues using the demonstrated protocol supplied by the manufacturer (10x Genomics, CG000632, Rev A). Briefly, three 50-µm sections were cut from FFPE blocks, deparaffinated with xylene (Merck) and rehydrated with an ethanol dilution row. After a wash step with PBS, 100 µl dissociation mix (1 mg ml^−1^ Liberase (Merck) in RPMI medium (Merck)) was added, and the samples were mechanically dissociated with a pellet pestle followed by enzymatic digestion at 37 °C for 30 min on a radial shaker (800 r.p.m.). Then the sample was triturated with a pipette and passed through a 30-µm filter. Cells were washed with tissue resuspension buffer (0.5 ml) and incubated with an antibody master mix containing anti-NeuN (clone 1B7, Alexa-647, Novus Biologicals) and anti-Olig2 (clone 211F1.1, Alexa-488, Merck). After two additional washes, single DAPI^+^NeuN^−^Olig2^−^ nuclei were sorted on a MoFlo Astrios machine (Beckman Coulter). From each sample, 200,000 nuclei were sorted into LoBind tubes (Eppendorf) and pooled into a control and transplanted sample, respectively.

The following steps were conducted following the 10x Genomics protocol entitled ‘Chromium Fixed RNA Profiling Reagent kits for Single-plexed Samples’ (CG000477 | Rev D). Briefly, sorted nuclei were resuspended in quenching buffer and centrifuged. The sorted pellet was resuspended in hybridization buffer containing Human WTA Probes BC001 (10x Genomics). After 20 h of hybridization at 42 °C the nuclei were repeatedly washed and counted using a hemocytometer. Up to 40,000 nuclei were loaded per reaction. Unused nuclei were stored at −80 °C in storage buffer (0.1 volume Enhancer in Post-Hyb Resuspension Buffer and 10% glycerol). A total of two pooled control and three pooled post-transplantation libraries were prepared from the same nuclei pools due to low yields.

### 10x Genomics droplet-based single-cell/single-nucleus library preparation

Up to 40,000 sorted cells or nuclei per reaction were loaded on a Chromium controller (10x Genomics). Complementary DNA amplification and library preparation were performed according to the user guide for the Chromium Next GEM Single Cell 3′ Reagent kits v.3.1 (CG000204 or CG000390). Additionally, the 3′ Feature Barcoding kit (10x Genomics) was used for library preparation of multiplexed and CITE-seq samples. The libraries were sequenced on a NextSeq 550 or NextSeq 1000/2000 machines (Illumina) with a sequencing depth appropriate to reach 20,000 reads per cell. The targeted sequencing depth for multiplexing and feature barcoding/CITE-seq libraries were 5,000 reads per cell for each modality. Transcriptome alignment to the GENCODE human genome release 33 was performed with CellRanger v.7.1.0 on a Linux workstation. The CellRanger multi workflow was applied for sample demultiplexing, transcript and surface protein quantification.

Fixed RNA-profiling libraries were generated using the protocol for single-plexed samples (CG000477). Reference probe set alignment and quantification was conducted using CellRanger v.7.1.0 on a Linux workstation with the CellRanger multi workflow.

### Integration and analysis of the 3′ mRNA 10x single-cell and single-nucleus transcriptome data

Data collection and analysis were performed in an unsupervised manner, but not blind to the conditions of the experiments. Filtered counts matrices were loaded with Seurat v.4.3.0 (ref. ^26^). Cells with at least 500 and fewer than 4,000 detected genes and below 20% mitochondrial transcripts were included. Doublet detection and removal were achieved using a combination of the scDblFinder v.1.10.0 and SingleCellExperiment v.1.18.1 packages.

The control 10x dataset consisted of samples with only transcriptome data and samples with transcriptome and CITE-seq information. The glioblastoma data contained transcriptome and CITE-seq information. We used the Azimuth algorithm^26^ to impute missing CITE-seq information. To this end, data from a reference experiment containing brain PC, LM and CP cells were normalized and scaled with 10,000 most-variable features using the SCTransform Seurat function. The other three control datasets were aligned to this reference using the FindNeighbors and FindTransferAnchors Seurat functions. Next, cell-surface-marker expression values were imputed using the MapQuery Seurat function.

For multimodal mapping, the transcriptome data from different experiments were merged into one Seurat object, then normalized and scaled on 10,000 most-variable features. Then, the different experiments within the Seurat object were integrated using the Harmony R package v.0.1.1 with ‘experiment’ as integration variable^59^. In the next step, the surface receptor data were normalized using a centered log ratio transformation as the normalization method and scaled on all available features. Dimensionality reduction was achieved using the RunPCA Seurat function. Then, multimodal mapping was conducted using the FindMultiModalNeighbors Seurat function with the top 30 components of the transcriptome and top 18 components of the surface receptor data. UMAP embedding was generated from the resulting weighted nearest-neighbor graph. Next, cell clusters were identified using the FindClusters Seurat function with the algorithm parameter set to the smart local moving algorithm and otherwise default settings.

### Analysis of the fixed mRNA-profiling single-nucleus data

A total of 9,035 nuclei with above 50 and below 1,000 transcripts were imported into a Seurat object. Doublet detection and removal were achieved using a combination of the scDblFinder v.1.10.0 and SingleCellExperiment v.1.18.1 packages. The data were normalized and scaled using the SCTransform function. After dimensionality reduction with RunPCA, UMAP embedding, nearest-neighbor identification and clustering were conducted from 30 principal components with default parameters.

### mCEL-Seq2 single-cell RNA amplification and library preparation

The following antibodies were used for FACS sorting: anti-CD45 (clone HI30, APC, BD Bioscience), anti-CD206 (also known as MRC1, clone 15-2, APC-Cy7, BioLegend), anti-CD3 (clone SP34-2, PE-Cy7, BD Bioscience), anti-CD19 (clone SJ25C1, PE-Cy7, BioLegend) and anti-CD20 (clone 2H7, PE-Cy7, BioLegend). On a MoFlo Astrios machine, CD45^+^CD206^+^Lin^−^ (CD3, CD19 and CD20) cells were sorted into 384-well plates (Bio-Rad Laboratories) (Extended Data Fig. 1b). scRNA-seq was conducted using the mCEL-Seq2 protocol^34,60^ on a mosquito nanoliter-scale liquid-handling robot (SPT Labtech). Eight libraries with 192 cells each were sequenced per lane on an Illumina HiSeq 3000 sequencing system (pair-end multiplexing run) at a depth of ~130,000–200,000 reads per cell.

Fastq files were aligned using STAR v.2.7.10a with default parameters to the human GENCODE human genome release 33 (ref. ^61^). The left read contains the barcode information; the first six bases represented the unique molecular identifier (UMI) followed by six bases with the cell specific barcode. A poly-T stretch comprised the remainder of the left read that was therefore not used for quantification.

### Analysis of the mCEL-Seq2 scRNA-seq data

Data collection and analysis were performed in an unsupervised manner, but not blind to the conditions of the experiments. Overall, 17 mCEL-Seq2 libraries were sequenced and after quality control, 2,490 cells were analyzed. Cells with at least 200 and fewer than 4,000 detected genes and below 20% mitochondrial transcripts were included. Doublet detection and removal were achieved using a combination of the scDblFinder v.1.10.0 and SingleCellExperiment v.1.18.1 packages. Data integration was performed using the Seurat v.4 algorithm running the multimodal reference mapping workflow with 10,000 features^26^. Briefly, data from each library were normalized and scaled using the SCTransform Seurat function. Then, each library was aligned to multimodal human peripheral blood mononuclear cell (PBMC) data as a reference using the FindNeighbors and FindTransferAnchors Seurat functions. We used PBMCs as a reference to confidently distinguish intravascular cells that are commonly present in human samples that cannot be perfused. This was necessary, as human cDC2 are expected in the CD206 gate due to known high *MRC1* expression in these cells^44^. The top 50 components of the supervised principal component dimensionality reduction were used to calculate the UMAP embedding and identify the nearest neighbors for cluster assignment. Clusters were calculated with the resolution parameter set to 0.3.

### Re-ordering of clusters

The default cluster ordering of Seurat is based on cluster size with the largest cluster first. To sort the clusters based on transcriptional similarity, we performed hierarchical clustering of the average gene expressions in each cluster. Similarly, cell types were ordered based on transcriptional similarity.

### Cell doublet identification and exclusion

Doublets were excluded using the scDblFinder package v.1.10.0. Briefly, the counts slot of the Seurat object was transformed into a SingleCellExperiment object, the scDblFinder function was run on the new object and the original Seurat object was filtered for cells classified as ‘singlet’.

### Cell type and state classification based on gene module expression scoring

Cell-cycle scoring and quantification of cell type and functional gene expression modules were conducted based on published gene signatures^28^ (Supplementary Table 21). Gene module scores were calculated using the UCell R package v.2.0.1 based on the Mann–Whitney *U* statistic^62^. Briefly, a cell type or cell state-associated gene list was passed to the ScoreSignatures_UCell function. The resulting cell-wise scores were added to the Seurat object using the AddMetadata Seurat function. Cell-cycle modules were scored in Seurat using the CellCycleScoring function, which is a wrapper for the AddModuleScore function. Low-quality cells were identified based on the percentage of mitochondrial across all genes and the expression of the *KCNQ1OT1* gene^43^.

Dendrograms showing the similarity of the identified cell types were prepared using Ward’s method for hierarchical clustering^63^.

### Reference-based cell-type assignment

Cell-type assignment was performed using the Azimuth algorithm^26^. Briefly, newly generated data were normalized and scaled using the SCTransform Seurat function. Then, they were aligned to multimodal human PBMC data as a reference using the FindNeighbors and FindTransferAnchors Seurat functions. The use of PBMCs as a reference was primarily chosen to confidently assign intravascular cells that are commonly present in human samples that cannot be perfused. The cell-type assignments were added to the original Seurat objects using the MapQuery Seurat function. Tissue-resident cell types not present in the dataset, such as CAMs or microglia were manually added based on the gene module expression of published marker genes (Supplementary Table 21)^7,27,32,44^. Also, transitory cells traversing from the blood vessels into the tissue were classified based on the expression of published cell-type homing-gene expression modules (Supplementary Table 21)^28^. To this end, the cluster-wise expression of the respective gene module calculated using the UCell package was assessed and the respective cell type was assigned accordingly.

Cell-type assignment in the fetal tissues was conducted using reference mapping with a subset of the Braun et al. dataset for PC/PV and Yang et al. dataset for CP cells^2,39^.

### Differential gene expression analysis

Differentially expressed genes were determined using the FindAllMarkers Seurat function with default settings. For side-by-side comparisons of clusters or conditions were achieved by running the FindMakers Seurat function with logfc.threshold = 0.01 and min.pct = 0.01.

### Cluster enrichment analysis

Hypergeometric testing with the phyper base R function was used to calculate enrichment of a given condition in a cluster based on the overall number of cells from this condition in the dataset. The calculated value stands for the probability that number *n* or more cells from a given condition are found in a cluster by chance. 0.05 was chosen as cutoff for statistical significance. The Benjamini–Hochberg method was used for multiple testing correction of the calculated *P* values for all conditions. The code for Marimekko plots was modified from R. Scavetta.

### MOFA2 latent factor analysis

Latent factors underlying the transcriptional differences between conditions were identified using the MOFA2 algorithm^31^. To this end, we adopted the ‘integration of a time-course single-cell RNA-seq dataset’ workflow. mCEL-Seq2 as specified under the following vignette: raw.githack.com/bioFAM/MOFA2_tutorials/master/R_tutorials/scRNA_gastrulation.html. Briefly, the cell types of interest were extracted from the Seurat object (CAMs, microglia and transitory monocytes (clusters 9 and 21) from the control and CAMs, microglia, TAMs and Trans. moTAMs from the tumor dataset, respectively). The data were normalized and rescaled. Then, the MOFA object was created from the RNA slot of the Seurat object and grouped by the compartments for the control data and composite variables consisting of the compartment and cell types for the tumor data. The convergence mode was set to fast. The number of latent factors was set to 10. After convergence of the MOFA model, the variance explained was visualized using the plot_variance_explained MOFA2 function. The weights for each latent factor were extracted using the get_weights function. The genes with the top 100 weights were extracted and comparative Gene Ontology analysis between the latent factors was performed using the compareCluster function from the clusterProfiler R package v.4.4.4 (ref. ^64^). The factor-wise Gene Ontology terms were visualized using the dot-plot function of the clusterProfiler package.

### Species comparison between human and mouse

For cross-species analysis, a previously published control CAMs dataset^7^ was analyzed using Seurat v.4 in an analogous way as the human data in the present study. Differentially expressed genes were separately assessed between CAMs and microglia in mice and humans using the FindMarkers Seurat function. Orthologous genes between human and murine samples were obtained using the biomaRt v.2.52.0 R package. For visualization, a scatter-plot shows the correlation of the average log_2_ fold change values between both datasets with positive values showing genes differentially expressed in CAMs and negative values for microglia genes. Human log_2_ fold change values are shown on the *x* axis and the murine ones are shown on the *y* axis.

### Pseudotime analysis

Pseudotime analysis was performed using the StemID analysis within the RaceID v.0.2.4 and FateID v.0.2.2 R packages^34,43^. We followed the vignette published by the package authors. Briefly, the gene the counts object of the Seurat object was transformed into a RaceID object. Then a lineage graph was calculated. Based on the suggested links a trajectory was chosen. Genes with at least one UMI in at least ten cells were included in the trajectory. Genes with a correlation coefficient of 0.85 or above were summarized into gene expression modules and modules of at least five cells were included in the analysis.

### Gene Ontology enrichment analysis

Gene Ontology term analysis was conducted using the clusterProfiler v.4.4.4 R package^64^. Cluster marker genes were obtained using the FindAllMarkers Seurat function. The top 25 of these markers were transformed into entrez IDs and passed to the compareCluster function of the clusterProfiler package.

### Data analysis and visualization

Data collection and analysis were performed in an unsupervised manner, but not blind to the conditions of the experiments. Data analysis was conducted using the R programming language v.4.0.2. The tidyverse R package was used for data processing and visualization (CRAN.R-project.org/package=tidyverse).

### Cell–cell interaction analysis

Cell–cell interaction analysis between different cell types in the single-nucleus RNA-seq samples was performed using the NICHES R package v.1.0.0 (ref. ^40^). following a published vignette at github.com/msraredon/NICHES/blob/master/vignettes/01%20NICHES%20Spatial.Rmd. Briefly, the combined immune-cell object containing published fetal PC/PV and postnatal CP immune cells^2,39^ was merged with the non-immune single-nucleus data from Fig. 5b. To account for the abundance of neuronal cell types in the non-immune dataset, it was downsampled to up to 500 distinct cells per cell type. The resulting Seurat object was passed to the RunNICHES function and the SystemToCell interactions were analyzed using the fantom5 ligand–receptor database. These data consist of the interactions between the respective cell types and all other cell types in the data. We were particularly interested in the differential interactions of pre- and postnatal cell macrophage subsets. To this end we ran differential expression analysis of ligand–receptor pairs using the FindMarkers R function and visualized to top differentially expressed pairs using volcano plots with the EnhancedVolcano R package v.1.14.0.

### In situ analysis using CARTANA technology

Four frozen OCT-embedded control and two glioblastoma samples were cryosectioned (10-μm thickness) and placed onto SuperFrost Plus glass slides (Thermo Fisher) and shipped on dry ice to CARTANA (part of 10x Genomics) for processing.

Samples were fixed (with 4% formaldehyde) permeabilized (with 0.1 mg ml^−1^ ± pepsin in 0.1 M HCl (P7012 Sigma-Aldrich)) before library preparation. For tissue section mounting, Slow Fade Antifade Mountant (Thermo Fisher) was used for optimal handling and imaging.

For library preparation, chimeric padlock probes (targeting directly RNA and containing an anchor sequence as well as a gene-specific barcode) for three predefined panels (CNS Glia, Immune General and Immune Oncology) as well as a custom panel of 50 genes (Supplementary Table 10 for all genes) were hybridized overnight at 37 °C, then ligated before the rolling circle amplification is performed overnight at 30 °C using the HS Library Preparation kit for CARTANA technology and following manufacturer’s instructions. All incubations were performed in SecureSealTM chambers (Grace Biolabs). Before final library preparation, optimal RNA integrity and assay conditions were assessed using Malat1 and Rplp0 housekeeping genes only using the same protocol.

Quality control of the library preparation was performed by applying anchor probes to detect simultaneously all rolling circle amplification products from all genes in all panels. Anchor probes are labeled probes with Cy5 fluorophore (excitation at 650 nm and emission at 670 nm).

All samples passed the quality control and were sent to CARTANA Sweden, for in situ barcode sequencing, imaging and data processing. Briefly, adaptor probes and sequencing pools (containing four different fluorescent labels: Alexa Fluor 488, Cy3, Cy5 and Alexa Fluor 750) were hybridized to the padlock probes to detect the gene-specific barcodes, through a sequence-specific signal for each gene-specific rolling circle amplification product. This was followed by imaging and performed six times in a row to allow for the decoding of all genes in the panel.

Raw data consisting of ×20 or ×40 images from five fluorescent channels (DAPI, Alexa Fluor 488, Cy3, Cy5 and Alexa Fluor 750) were each taken as a *z* stack and flattened to two dimensions using maximum intensity projection. After image processing and decoding, the results were summarized in a csv file and gene plots were generated using MATLAB.

Image segmentation of the ISS data was performed with the docker image of the Baysor tool (v.0.4.2.)^65^, using the following modified parameters for the configuration file: min-molecules-per-cell,10; scale, 50; scale-std, 50%; and min-molecules-per-segment, 2. The Baysor analysis was also run using a prior segmentation performed in ImageJ Fiji. The segmentation was performed with the watershed algorithm on an DAPI image indicating nuclei position, using local thresholding when needed. The parameter value used for the prior segmentation confidence was 0.8.

### Analysis of the in situ sequencing data

The cell counts matrix and the cell coordinates obtained from image segmentation analysis with Baysor were loaded into the SPAtial Transcriptomic Analysis R package (SPATA2) v.0.1.0. with the initiateSpataObject_CountMtr function. Tissue segmentation of anatomical areas in control samples was anatomically drawn in the SPATA2 viewer after calling the createSegmentation function. For hypoxic areas in the tumor, we visualized in the gene set RCTM_CELLULAR_RESPONSE_TO_HYPOXIA in the SPATA2 viewer. Then, the segments were manually drawn around areas with high expression of the gene set. Similarly, spatial trajectory analysis was performed using the createTrajectories function and manually drawing a trajectory from an area of cellular tumor into the hypoxic area. The top ten up- and downregulated genes along the trajectory were identified using the assessTrajectoryTrends and extracted using the filterTrajectoryTrends functions. Visualization of the resulting smoothed gene expressions was conducted using the plotTrajectoryHeatmap function. Representative genes were visualized using the plotSurfaceComparison function.

Cell-type classification was conducted using label transfer via the Azimuth algorithm. Briefly, the SPATA2 object was converted into a Seurat object using the transformSpataToSeurat function. The data were normalized and scaled on all available genes. For the control samples, LM and PC/PV cells were separately classified with single-nucleus RNA-seq datasets from the LM and PC/PV. The reference datasets were subset to the genes contained in the ISS panel and nuclei with more than ten transcripts were kept. Then, the ISS dataset was integrated with the reference dataset using the FindTransferAnchors Seurat function with the dims parameter set to 1:10. Cell-type labels were transferred using the TransferData with the dims parameter set to 1:10. For the glioblastoma samples, the strategy was slightly adjusted. To account for the predominance of tumor cells in the reference single-nucleus sequencing dataset, it was downsampled to up to 200 distinct cells per cell type. Then we ran the FindTransferAnchors and TransferData Seurat functions with dims set to 1:30. The cell-type classifications were controlled based on differentially expressed genes and manually adjusted, if needed.

### Spatial neighborhood analysis

Spatial neighborhood enrichment analysis was performed using the Squidpy algorithm v.1.3.0 (ref. ^36^) as previously described (www.sc-best-practices.org/spatial/neighborhood.html). Neighborhood enrichment scores of the respective cell types were calculated using the nhood_enrichment function. Subsequently, the interaction_matrix function was used to obtain spatial interaction scores of the cell types. Plotting was performed using Scanpy v.1.9.3 (ref. ^66^) functionality.

### CosMx tissue processing, image acquisition, cell segmentation and data analysis for 1,000-plex RNA profiling

FFPE tissue blocks with control and tumor samples were sectioned to consecutive 5-µm slices using a microtome and shipped to NanoString Technologies for processing. Sample processing, staining, imaging and cell segmentation were performed as previously described^67^. Briefly, tissue sections were placed onto VWR Superfrost Plus Micro slides for optimal adherence. Slides were then dried at 37 °C overnight, followed by deparaffinization, antigen retrieval and proteinase-mediated permeabilization (nanostring.com/products/cosmx-spatial-molecular-imager/single-cell-imaging-overview). Then,1 nM RNA-ISH probes were applied for hybridization at 37 °C overnight. After a stringent wash, a flow cell was assembled on top of the slide and cyclic RNA readout on CosMx was performed (16-digit encoding strategy). After all cycles were completed, additional visualization markers for morphology and cell segmentation were added, including pan-cytokeratin, CD45, CD3, CD298/B2M and DAPI. Twenty-four 0.985 mm × 0.657 mm fields of view (FOVs) were selected for data collection in each slice. The CosMx optical system has an epifluorescent configuration based on a customized water objective (×13, NA 0.82) and uses widefield illumination with a mix of lasers and light-emitting diodes (385 nm, 488 nm, 530 nm, 590 nm and 647 nm) that allow imaging of DAPI, Alexa Fluor 488, Atto-532, Dyomics Dy-605 and Alexa Fluor 647, as well as removal of photocleavable dye components. The camera was a FLIR BFS-U3_200S6M-C based on the IMX183 Sony industrial CMOS sensor (pixel size 180 nm). A three-dimensional multichannel image stack (nine frames) was obtained at each FOV location, with a step size of 0.8 µm. Registration, feature extraction, localization, decoding of the presence individual transcripts and machine-learning-based cell segmentation (developed upon Cellpose) were performed as previously described^67^. The final segmentation mapped each transcript in the registered images to the corresponding cell, as well as to subcellular compartments (nuclei, cytoplasm and membrane), where the transcript is located.

Tissue segmentation and trajectory analysis was conducted similarly to the ISS data described above using the SPATA2 R package. Also, cell-type classification was conducted with the reference single-nucleus sequencing datasets subset to the genes contained in the 1,000-plex gene panel.

### Spatially resolved cell–cell interaction analysis

Spatially resolved cell–cell interaction analysis between different cell types was performed on the GeoMx data using the NICHES R package v.1.0.0. following a published vignette at github.com/msraredon/NICHES/blob/master/vignettes/01%20NICHES%20Spatial.Rmd. Briefly, missing gene expression values were imputed using the Adaptively-thresholded Low Rank Approximation algorithm on the Seurat object^68^. The resulting Seurat object was passed to the RunNICHES function and the NeighborhoodToCell interactions were analyzed using the fantom5 ligand–receptor database. These data consist of the interaction between the respective cell types in the data and their neighboring cells. The differentially expressed interactions were visualized.

### Human sex-mismatched PBSCT autopsy cases

All human brain autopsy samples were derived from the case archive of the Institute of Neuropathology, University of Freiburg. Brains and DM of autopsy cases were transferred into 4% paraformaldehyde within less than 48 h after death and fixed for at least 1 week. After fixation, representative tissue from several brain regions of the left hemisphere was dissected and embedded in paraffin. Among the 15 female patients with a history of male donor-derived PBSCT, paraffin-embedded samples the frontal cortex and cerebellum were available in 14 cases and the hippocampus was available in 13 cases. Hippocampal samples derived from nine patients contained parts of the CP. In nine cases, LM were amenable for analysis as they were well preserved and confounding neuropathological diagnosis was absent. In five female sex-mismatched PBSCT patients and four control cases, the DM adjacent to the frontal branch of the medial meningeal artery was resected and processed for further histological analysis.

### Chromogenic in situ hybridization of human brain samples

To investigate CAM engraftment, IHC and CISH labeling the Y chromosome in samples of female sex-mismatched PBSCT patients was performed using 10-µm thick sections as previously described^42^. Briefly, IHC was carried out using Liquid Permanent Red Substrate-Chromogen (Agilent Dako) for antigen visualization. Subsequently, CISH was performed using the ZytoDot CISH Implementation kit (ZytoVision) according to the manufacturer’s instructions with the following modifications. Sections were incubated in EDTA at 95 °C for 15 min and treated with pepsin solution at 37 °C for 6 min. After dehydration, 12 µl of ZytoDot CEN Yq12 digoxigenin-linked probe (ZytoVision) was added for at least 20 h at 37 °C. After washing and blocking steps, sections were incubated in mouse anti-digoxigenin antibody solution, treated with HRP-conjugated anti-mouse antibody at 37 °C for 30 min and bound to DAB at 37 °C for 45 min. Nuclei were counterstained with hematoxylin.

### Engraftment analysis

Sections were analyzed using a MikroCam II with a UPlan FLN ×40/0.75 NA objective on a BX40 microscope (Olympus). To scan for the Y chromosome the focus plain was carefully moved through the whole nucleus of each cell analyzed.

First, the detection rate of Y chromosomes was determined for each CAM compartment in *n* = 3 male control samples. For each compartment and marker, several FOVs were analyzed. The mean Y chromosome detection rate for each CNS myeloid cell niche and marker was calculated.

Next, samples derived from female sex-mismatched PBSCT cases were analyzed. In each compartment, at least 50 CAMs per patient were analyzed. Myeloid cell engraftment rates were calculated by dividing the resulting percentage of Y^+^ cells in each compartment by the corresponding Y chromosome detection rate previously determined in male control tissue.

*T*_50_, the duration after PBSCT required for a turnover of 50% of CAMs by donor-derived cells, was inferred by a linear regression analysis between log_10_-transformed time interval after PBSCT and Pearson’s correlation coefficient. The values for the confidence intervals were added based on the estimated upper and lower bounds of the correlation coefficient.

### Assessment of marker regulation in donor- and host-derived cells

To investigate expression of CAM markers in donor-derived cells of the LM and PV space, three patients with long survival after PBSCT and consecutively high numbers of engrafting donor-derived cells were chosen. In these patients, at least 100 perivascular and leptomeningeal Y^+^ cells pooled from cortical, hippocampal and cerebellar samples were assessed for Iba1 (Abcam, clone EPR 16588), CD206 (Abnova, clone 5C11) and Siglec1 expression. For microglia quantification, cortical samples were assessed for Iba1 (Abcam, clone EPR 16588), P2RY12 (Sigma-Aldrich, polyclonal), TMEM119 (Abcam, polyclonal) and GLUT5 (Sigma-Aldrich, polyclonal).

### Intracellular barcoding for mass cytometry

Percoll-isolated myeloid cells were fixed with fixation/stabilization buffer^69^ (Smart Tube) and frozen at −80 °C until analysis by mass cytometry. Cells were thawed and subsequently stained with premade combinations of six different palladium isotopes: 102 Pd, 104 Pd, 105 Pd, 106 Pd, 108 Pd and 110 Pd (Cell-ID 20-plex Pd Barcoding kit, Fluidigm). This multiplexing kit applies a 6-choose-3 barcoding scheme that results in 20 different combinations of three Pd isotopes. After 30 min staining (at RT), individual samples were washed twice with cell staining buffer (0.5% bovine serum albumin in PBS, containing 2 mM EDTA). All samples were pooled together, washed and further stained with antibodies.

### Antibodies

Anti-human antibodies (Supplementary Table 9) were purchased either preconjugated to metal isotopes (Fluidigm) or from commercial suppliers in purified form and conjugated in-house using the MaxPar X8 kit (Fluidigm) according to the manufacturer’s protocol. For surface and intracellular staining, after cell barcoding, washing and pelleting, the combined samples were resuspended in 100 µl antibody cocktail against surface markers (Supplementary Table 9) and incubated for 30 min at 4 °C. Then, cells were washed twice with cell staining buffer. For intracellular staining, the stained (non-stimulated) cells were then incubated in fixation/permeabilization buffer (Fix/Perm Buffer, eBioscience) for 60 min at 4 °C. Cells were then washed twice with permeabilization buffer (eBioscience). The samples were then stained with antibody cocktails against intracellular molecules in permeabilization buffer for 1 h at 4 °C. Cells were subsequently washed twice with permeabilization buffer and incubated overnight in 2% methanol-free formaldehyde solution. Fixed cells were then washed and resuspended in 1 ml iridium intercalator solution (Fluidigm) for 1 h at RT. Next, the samples were washed twice with cell staining buffer and then twice with ddH_2_O (Fluidigm). Cells were pelleted and kept at 4 °C until CyTOF measurement.

### CyTOF measurement

Cells were analyzed using a CyTOF2 upgraded to Helios specifications, with software v.6.5.236. The instrument was tuned according to the manufacturer’s instructions with tuning solution (Fluidigm) and measurement of EQ four element calibration beads (Fluidigm) containing 140/142Ce, 151/153Eu, 165Ho and 175/176Lu served as a quality control for sensitivity and recovery. Directly before analysis, cells were resuspended in ddH_2_O, filtered (20 µm Celltrix, Sysmex), counted and adjusted to 3–5 × 10^5^ cells ml^−1^. EQ four element calibration beads were added at a final concentration of 1:10 of the sample volume to be able to normalize the data to compensate for signal drift and day-to-day changes in instrument sensitivity.

Samples were acquired with a flow rate of 300–400 events s^−1^. The lower convolution threshold was set to 400, with noise reduction mode on and cell definition parameters set at event duration of 10–150. The resulting flow cytometry standard (FCS) files were normalized and randomized using the CyTOF software’s internal FCS-Processing module on the non-randomized (‘original’) data. Settings were used according to the default settings in the software with time interval normalization (100 s per minimum of 50 beads) and passport v.2. Intervals with fewer than 50 beads per 100 s were excluded from the resulting fcs file.

### Mass cytometry data processing and analysis

Cytobank was used for initial manual gating on intact single cells. Nucleated single cells were manually gated by DNA intercalators ^191^Ir/^193^Ir and event length. For de-barcoding, Boolean gating was used to deconvolute individual sample according to the barcode combination. For gated cells from different individuals, expression levels of each marker were assessed and visualized in dot plots and/or histograms. After de-barcoding, each sample was exported as individual .fcs file from Cytobank. We performed the visualization and clustering analysis of the data using the *k*-nearest-neighbor density-based algorithm X-shift on the VorteX Clustering Environment (github.com/nolanlab/vortex/).

### Data analysis and visualization

Data analysis and visualization was mainly conducted in the R v.4.2.0 programming environment using the tidyverse package suite v.2.0.0. and specialized visualization packages, including ComplexHeatmap v.2.12.1 (refs. ^70–73^).

### Statistics and reproducibility

No statistical method was used to predetermine sample size. The experiments were not randomized. The investigators were blinded during the analysis of microscopy analyses. For single-cell and single-nucleus RNA-seq, cells with fewer than 500 and more than 4,000 detected genes and more than 20% mitochondrial transcripts were excluded. The presented CITE-seq data only contain cells with more than 50 counts per cell. For Cel-Seq2 data, cells with fewer than 200 and more than 4,000 detected genes and more than 20% mitochondrial transcripts were excluded. Furthermore, clusters with biologically uninformative low-quality cells were excluded after evaluation. The exclusion criteria were in line with previously applied strategies for Cel-Seq2 data^32,43^. For fixed RNA-profiling samples, cells with fewer than 50 and more than 1,000 detected genes were excluded. ISS data only contain cells with five or more counts per cell.

### Reporting summary

Further information on research design is available in the Nature Portfolio Reporting Summary linked to this article.

## Online content

Any methods, additional references, Nature Portfolio reporting summaries, source data, extended data, supplementary information, acknowledgements, peer review information; details of author contributions and competing interests; and statements of data and code availability are available at 10.1038/s41591-023-02673-1.

### Supplementary information

Reporting Summary

Supplementary Tables 1–21 **Supplementary Table 1**. Cell metadata of the control CD45^+^ dataset. **Supplementary Table 2**. Results of the one-sided hypergeometric testing of the cluster-wise compartment contributions. **Supplementary Table 3**. Top 20 cluster marker genes of the control CD45^+^ dataset. Significance was calculated using two-sided unpaired Wilcoxon rank-sum tests followed by Bonferroni correction for multiple testing. **Supplementary Table 4**. Top 100 genes per latent factor identified by MOFA2 analysis. **Supplementary Table 5**. Genes contained in the species comparison between human and mouse cells in Extended Data Fig. 3c. Statistical testing was performed by two-sided hypergeometric tests followed by adjustment for multiple testing using the Benjamini–Hochberg method. **Supplementary Table 6**. Results of the one-sided hypergeometric testing of the cluster-wise cell type contributions of cells from the CD45^+^CD206^+^Lin^−^ gate of CNS border regions analyzed using mCEL-Seq2. Statistical testing was performed by two-sided hypergeometric tests followed by adjustment for multiple testing using the Benjamini–Hochberg method. **Supplementary Table 7**. Cluster marker genes of cells from the CD45^+^CD206^+^Lin^−^ gate of CNS border regions analyzed using mCEL-Seq2. Significance was calculated using two-sided unpaired Wilcoxon rank-sum tests followed by Bonferroni correction for multiple testing. **Supplementary Table 8**. Cluster marker profiles for CITE-seq data of control cells from Fig. 2e. Significance was calculated using two-sided unpaired Wilcoxon rank-sum tests followed by Bonferroni correction for multiple testing. **Supplementary Table 9**. Mass cytometry antibody panel. **Supplementary Table 10**. ISS gene panel. **Supplementary Table 11**. Nanostring CosMx gene panel. **Supplementary Table 12**. Top 20 cluster marker genes of the fetal single-nucleus sequencing dataset. Significance was calculated using two-sided unpaired Wilcoxon rank-sum tests followed by Bonferroni correction for multiple testing. **Supplementary Table 13**. Up to top 100 marker genes for pre and postnatal CAMs, microglia and Kolmer cells. Significance was calculated using two-sided unpaired Wilcoxon rank-sum tests followed by Bonferroni correction for multiple testing. **Supplementary Table 14**. Genes visualized in the pseudotime analysis heat map in Fig. 6c. **Supplementary Table 15**. Up to top 10 cluster marker genes of myeloid cells analyzed with single-nucleus RNA profiling in FFPE samples. Significance was calculated using two-sided unpaired Wilcoxon rank-sum tests followed by Bonferroni correction for multiple testing. **Supplementary Table 16**. Results of the one-sided hypergeometric testing of the cluster-wise contributions of cells from control and tumor tissues. **Supplementary Table 17**. Top 20 cluster marker genes of the tumor dataset. Statistical testing was performed by two-sided hypergeometric tests followed by adjustment for multiple testing using the Benjamini–Hochberg method. **Supplementary Table 18**. Results of the one-sided hypergeometric testing of the cluster-wise contributions of cells from LM and PC/PV. **Supplementary Table 19**. Top 100 genes per latent factor identified by MOFA2 analysis in Fig. 6e. **Supplementary Table 20**. Characteristics of the patient samples analyzed in the present study. **Supplementary Table 21**. Marker genes for cell types and other gene expression modules.

### Source data

Source Data Fig. 1 Numeric tables and statistical source data.

Source Data Fig. 2 Numeric tables and statistical source data.

Source Data Fig. 3 Numeric tables and statistical source data.

Source Data Fig. 4 Numeric tables and statistical source data.

Source Data Fig. 5 Numeric tables and statistical source data.

Source Data Fig. 6 Numeric tables and statistical source data.

Source Data Extended Data Fig. 2 Numeric tables and statistical source data.

Source Data Extended Data Fig. 3 Numeric tables and statistical source data.

Source Data Extended Data Fig. 4 Numeric tables and statistical source data.

Source Data Extended Data Fig. 6 Numeric tables and statistical source data.

Source Data Extended Data Fig. 7 Numeric tables and statistical source data.

Source Data Extended Data Fig. 8 Numeric tables and statistical source data.

## Data Availability

The processed data for this project are available under GSE245311. The raw sequencing files are access-restricted and can be accessed at the European Genome–phenome Archive under the accession number EGAS50000000030 (https://ega-archive.org/studies/EGAS50000000030). Access to the data will require a Data Transfer Agreement. The raw data for the mass cytometry experiments can be found under flow repository ID FR-FCM-Z6S6. Published counts data^26^ for reference mapping of the immune cells were downloaded from atlas.fredhutch.org/data/nygc/multimodal/pbmc_multimodal.h5seurat. Published counts data^2^ for control human CP single-nucleus RNA-seq samples were downloaded under the accession code GSE159812. Published counts^39^ data for reference mapping and comparative analyses for prenatal immune cells were downloaded from github.com/linnarsson-lab/developing-human-brain/. Published counts data^44^ for comparative analyses of the glioblastoma samples were downloaded from www.brainimmuneatlas.org/data_files/toDownload/filtered_feature_bc_matrix_HumanGBMciteSeq.zip and their metadata are at www.brainimmuneatlas.org/data_files/toDownload/annot_Human_TAM_DC_Mono_citeSeq.csv. Source data are provided with this paper.

## Code availability

The computer code for this project can be found and explored at www.brain-immunity.de.
