# Supplementary material for: Multiomic spatial landscape of innate immune cells at human central nervous system borders
Source: Nat Med. 2023 Dec 20;30(1):186–98. doi: 10.1038/s41591-023-02673-1 (PMC10803260; doi:10.1038/s41591-023-02673-1)
Supplement: Supplementary file 1 — Reporting Summary [file 41591_2023_2673_MOESM1_ESM.pdf]

nature portfolio

Corresponding author(s): Prof. Dr. Marco Prinz
Dr. Roman Sankowski

Last updated by author(s): Jun 15, 2023

Reporting Summary

Nature Portfolio wishes to improve the reproducibility of the work that we publish. This form provides structure for consistency and transparency in reporting. For further information on Nature Portfolio policies, see our Editorial Policies and the Editorial Policy Checklist.

Statistics

For all statistical analyses, confirm that the following items are present in the figure legend, table legend, main text, or Methods section.

n/a	Confirmed	
☐	☒	The exact sample size (*n*) for each experimental group/condition, given as a discrete number and unit of measurement
☐	☒	A statement on whether measurements were taken from distinct samples or whether the same sample was measured repeatedly
☐	☒	The statistical test(s) used AND whether they are one- or two-sided<br>*Only common tests should be described solely by name; describe more complex techniques in the Methods section.*
☒	☐	A description of all covariates tested
☐	☒	A description of any assumptions or corrections, such as tests of normality and adjustment for multiple comparisons
☐	☒	A full description of the statistical parameters including central tendency (e.g. means) or other basic estimates (e.g. regression coefficient) AND variation (e.g. standard deviation) or associated estimates of uncertainty (e.g. confidence intervals)
☐	☒	For null hypothesis testing, the test statistic (e.g. *F*, *t*, *r*) with confidence intervals, effect sizes, degrees of freedom and *P* value noted<br>*Give P values as exact values whenever suitable.*
☒	☐	For Bayesian analysis, information on the choice of priors and Markov chain Monte Carlo settings
☒	☐	For hierarchical and complex designs, identification of the appropriate level for tests and full reporting of outcomes
☐	☒	Estimates of effect sizes (e.g. Cohen's *d*, Pearson's *r*), indicating how they were calculated

Our web collection on statistics for biologists contains articles on many of the points above.

Software and code

Policy information about availability of computer code

Data collection	RNA sequencing:<br>NextSeq 1000/2000 Control Software (NCS) v1.2.0.36376 for the Illumina NextSeq 1000/2000 instrument<br>Illumina HiSeq 3000: HiSeq Control Software 2.0.2, RTA 2.4.11 / Recipe Fragment 2.0.0.2<br>bcl2fastq v2.20 software for conversion of .bcl files into fastq files<br>DRAGEN v3.8.4 software for sequencing and conversion of .bcl files into fastq files<br><br>STAR (version v2.7.10a) for transcriptome aligment of paired end reads (scRNA-seq) to the GENCODE human genome release 33.<br><br>cellranger-7.1.0 was used with the GENCODE human genome release 33 human reference genome to align 10X samples.<br><br>Microscopy:<br>FV10-ASW Ver.4.2a was used for confocal imaging with the Olympus FV 1000<br><br>Leica Application Suite (LAS) X 3.5.7.23225 was used for confocal imaging with the Leica TCS SP8 X
Data analysis	R version 4.2.0 (2022-04-22)<br>Platform: x86_64-apple-darwin17.0 (64-bit)<br>Running under: macOS 13.3.1<br><br>Matrix products: default<br>LAPACK: /Library/Frameworks/R.framework/Versions/4.2/Resources/lib/libRlapack.dylib

```
locale:
[1] en_US.UTF-8/en_US.UTF-8/en_US.UTF-8/C/en_US.UTF-8/en_US.UTF-8

attached base packages:
[1] grid      stats4    stats     graphics  grDevices utils     datasets  methods   base

other attached packages:
 [1] biomaRt_2.52.0             harmony_0.1.1              Rcpp_1.0.10
 [4] FSA_0.9.4                  data.table_1.14.8          scDblFinder_1.10.0
 [7] SingleR_1.10.0             scCustomize_1.1.1          cowplot_1.1.1
[10] stxBrain.SeuratData_0.1.1  ssHippo.SeuratData_3.1.4   SeuratData_0.2.2
[13] NICHES_1.0.0               SeuratWrappers_0.3.0       edgeR_3.38.4
[16] limma_3.54.2               uwot_0.1.14                scran_1.24.1
[19] scater_1.24.0              scuttle_1.6.3              SingleCellExperiment_1.18.1
[22] SummarizedExperiment_1.26.1 Biobase_2.58.0            GenomicRanges_1.48.0
[25] GenomeInfoDb_1.32.4        clusterProfiler_4.4.4      MOFAdata_1.12.0
[28] circlize_0.4.15            ComplexHeatmap_2.12.1      HDF5Array_1.24.2
[31] rhdf5_2.40.0               DelayedArray_0.24.0        IRanges_2.32.0
[34] S4Vectors_0.36.2           MatrixGenerics_1.10.0      matrixStats_0.63.0
[37] BiocGenerics_0.44.0        Matrix_1.5-4               MOFA2_1.6.0
[40] viridis_0.6.2              viridisLite_0.4.1          forcats_1.0.0
[43] stringr_1.5.0              dplyr_1.1.1                purrr_1.0.1
[46] readr_2.1.4                tidyr_1.3.0                tibble_3.2.1
[49] tidyverse_2.0.0            tidyquant_1.0.7            quantmod_0.4.22
[52] TTR_0.24.3                 PerformanceAnalytics_2.0.4 xts_0.13.0
[55] zoo_1.8-11                 lubridate_1.9.2            assertthat_0.2.1
[58] SeuratDisk_0.0.0.9020      SeuratObject_4.1.3         Seurat_4.3.0
[61] rstatix_0.7.2              ggpubr_0.6.0               UCell_2.0.1
[64] EnhancedVolcano_1.14.0     ggrepel_0.9.3              ggplot2_3.4.2
RaceID_0.3.3        FateID_0.2.2          SPATA2  emmeans_1.8.8

loaded via a namespace (and not attached):
  [1] rsvd_1.0.5                ica_1.0-3                 Rsamtools_2.12.0
  [4] foreach_1.5.2             lmtest_0.9-40             crayon_1.5.2
  [7] MASS_7.3-58.3             rhdf5filters_1.8.0        nlme_3.1-162
 [10] backports_1.4.1           basilisk_1.8.1            GOSemSim_2.22.0
 [13] rlang_1.1.0               XVector_0.36.0            ROCR_1.0-11
 [16] irlba_2.3.5.1             filelock_1.0.2            xgboost_1.7.5.1
 [19] BiocParallel_1.30.4       rjson_0.2.21              bit64_4.0.5
 [22] glue_1.6.2                pheatmap_1.0.12           sctransform_0.3.5
 [25] parallel_4.2.0            vipor_0.4.5               spatstat.sparse_3.0-1
 [28] AnnotationDbi_1.58.0      DOSE_3.22.1               spatstat.geom_3.1-0
 [31] tidyselect_1.2.0          fitdistrplus_1.1-8        XML_3.99-0.14
 [34] GenomicAlignments_1.32.1  xtable_1.8-4              magrittr_2.0.3
 [37] cli_3.6.1                 zlibbioc_1.42.0           rstudioapi_0.14
 [40] miniUI_0.1.1.1            sp_1.6-0                  fastmatch_1.1-3
 [43] treeio_1.20.2             shiny_1.7.4               BiocSingular_1.12.0
 [46] clue_0.3-64               cluster_2.1.4             tidygraph_1.2.3
 [49] KEGGREST_1.36.3           ape_5.7-1                 listenv_0.9.0
 [52] Biostrings_2.64.1         png_0.1-8                 future_1.32.0
 [55] withr_2.5.0               bitops_1.0-7              ggforce_0.4.1
 [58] plyr_1.8.8                dqrng_0.3.0               pillar_1.9.0
 [61] GlobalOptions_0.1.2       cachem_1.0.7              hdf5r_1.3.8
 [64] GetoptLong_1.0.5          paletteer_1.5.0           DelayedMatrixStats_1.20.0
 [67] vctrs_0.6.1               ellipsis_0.3.2            generics_0.1.3
 [70] tools_4.2.0               beeswarm_0.4.0            munsell_0.5.0
 [73] tweenr_2.0.2              fgsea_1.22.0              rtracklayer_1.56.1
 [76] fastmap_1.1.1             compiler_4.2.0            abind_1.4-5
 [79] httpuv_1.6.9              plotly_4.10.1             GenomeInfoDbData_1.2.8
 [82] gridExtra_2.3             lattice_0.21-8            dir.expiry_1.4.0
 [85] deldir_1.0-6              utf8_1.2.3                later_1.3.0
 [88] BiocFileCache_2.4.0       Quandl_2.11.0             jsonlite_1.8.4
 [91] scales_1.2.1              ScaledMatrix_1.4.1        tidytree_0.4.2
 [94] pbapply_1.7-0             carData_3.0-5             sparseMatrixStats_1.10.0
 [97] lazyeval_0.2.2            promises_1.2.0.1          car_3.1-2
[100] doParallel_1.0.17         R.utils_2.12.2            goftest_1.2-3
[103] spatstat.utils_3.0-2      reticulate_1.28           statmod_1.5.0
[106] Rtsne_0.16                downloader_0.4            igraph_1.4.2
[109] yaml_2.3.7                survival_3.5-5            htmltools_0.5.5
[112] memoise_2.0.1             BiocIO_1.6.0              locfit_1.5-9.7
[115] graphlayouts_0.8.4        quadprog_1.5-8            digest_0.6.31
[118] mime_0.12                 rappdirs_0.3.3            RSQLite_2.3.1
[121] yulab.utils_0.0.6         future.apply_1.10.0       remotes_2.4.2
[124] blob_1.2.4                R.oo_1.25.0               splines_4.2.0
[127] rematch2_2.1.2            Rhdf5lib_1.18.2           RCurl_1.98-1.12
```

```
[130] broom_1.0.4             hms_1.1.3               colorspace_2.1-0
[133] BiocManager_1.30.20     ggbeeswarm_0.7.1        shape_1.4.6
[136] aplot_0.1.10            ggrastr_1.0.1           RANN_2.6.1
[139] enrichplot_1.16.2       fansi_1.0.4             tzdb_0.3.0
[142] parallelly_1.35.0       R6_2.5.1                ggridges_0.5.4
[145] lifecycle_1.0.3         bluster_1.6.0           curl_5.0.0
[148] ggsignif_0.6.4          leiden_0.4.3            snakecase_0.11.0
[151] DO.db_2.9               qvalue_2.28.0           RcppAnnoy_0.0.20
[154] RColorBrewer_1.1-3      iterators_1.0.14        spatstat.explore_3.1-0
[157] htmlwidgets_1.6.2       beachmat_2.12.0         polyclip_1.10-4
[160] shadowtext_0.1.2        timechange_0.2.0        gridGraphics_0.5-1
[163] globals_0.16.2          patchwork_1.1.2         spatstat.random_3.1-4
[166] progressr_0.13.0        codetools_0.2-19        metapod_1.4.0
[169] GO.db_3.15.0            prettyunits_1.1.1       dbplyr_2.3.2
[172] basilisk.utils_1.8.0    R.methodsS3_1.8.2       gtable_0.3.3
[175] DBI_1.1.3               ggfun_0.0.9             tensor_1.5
[178] httr_1.4.5              KernSmooth_2.23-20      stringi_1.7.12
[181] progress_1.2.2          reshape2_1.4.4          farver_2.1.1
[184] ggtree_3.4.4            xml2_1.3.3              BiocNeighbors_1.14.0
[187] restfulr_0.0.15         ggplotify_0.1.0         scattermore_0.8
[190] bit_4.0.5               scatterpie_0.1.8        spatstat.data_3.0-1
[193] janitor_2.2.0           ggraph_2.1.0            pkgconfig_2.0.3
[196] ggprism_1.0.4           corrplot_0.92

Python v3.10.12 environment:
aiobotocore==2.5.4
aiohttp==3.8.5
aioitertools==0.11.0
aiosignal==1.3.1
anndata==0.9.2
anyio==3.7.1
appnope==0.1.3
argon2-cffi==23.1.0
argon2-cffi-bindings==21.2.0
arrow==1.2.3
asciitree==0.3.3
asttokens==2.2.1
async-lru==2.0.4
async-timeout==4.0.3
attrs==23.1.0
awscli @ file:///Users/runner/miniforge3/conda-bld/awscli_1690854462982/work
Babel==2.12.1
backcall==0.2.0
beautifulsoup4==4.12.2
bleach==6.0.0
boltons @ file:///home/conda/feedstock_root/build_artifacts/boltons_1677499911949/work
botocore==1.31.17
Brotli @ file:///Users/runner/miniforge3/conda-bld/brotli-split_1687884292427/work
brotlipy @ file:///Users/runner/miniforge3/conda-bld/brotlipy_1666764759924/work
certifi==2023.7.22
cffi @ file:///Users/runner/miniforge3/conda-bld/cffi_1671179893800/work
charset-normalizer @ file:///home/conda/feedstock_root/build_artifacts/charset-normalizer_1688813409104/work
click==8.1.7
click-plugins==1.1.1
cligj==0.7.2
cloudpickle==2.2.1
colorama @ file:///home/conda/feedstock_root/build_artifacts/colorama_1602866480661/work
colorcet==3.0.1
comm==0.1.4
conda==23.3.1
conda-package-handling @ file:///home/conda/feedstock_root/build_artifacts/conda-package-handling_1669907009957/work
conda_package_streaming @ file:///home/conda/feedstock_root/build_artifacts/conda-package-streaming_1685101166527/work
contourpy==1.1.0
cryptography @ file:///Users/runner/miniforge3/conda-bld/cryptography-split_1689056134643/work
cycler==0.11.0
dask==2023.8.1
dask-image==2023.8.1
datashader==0.15.2
datashape==0.5.2
debugpy==1.6.7.post1
decorator==5.1.1
defusedxml==0.7.1
distributed==2023.8.1
docrep==0.3.2
docutils @ file:///Users/runner/miniforge3/conda-bld/docutils_1667993929972/work
entrypoints==0.4
```

```
exceptiongroup==1.1.3
executing==1.2.0
fasteners==0.18
fastjsonschema==2.18.0
Fiona==1.9.4.post1
fonttools==4.42.1
fqdn==1.5.1
frozenlist==1.4.0
fsspec==2023.6.0
geopandas==0.13.2
h5py==3.9.0
idna @ file:///home/conda/feedstock_root/build_artifacts/idna_1663625384323/work
igraph==0.10.6
imageio==2.31.1
importlib-metadata==6.8.0
inflect==7.0.0
ipykernel==6.25.1
ipython==8.14.0
isoduration==20.11.0
jedi==0.19.0
Jinja2==3.1.2
jmespath @ file:///home/conda/feedstock_root/build_artifacts/jmespath_1655568249366/work
joblib==1.3.2
json5==0.9.14
jsonpatch @ file:///home/conda/feedstock_root/build_artifacts/jsonpatch_1632759296524/work
jsonpointer==2.0
jsonschema==4.19.0
jsonschema-specifications==2023.7.1
jupyter-events==0.7.0
jupyter-lsp==2.2.0
jupyter_client==8.3.0
jupyter_core==5.3.1
jupyter_server==2.7.2
jupyter_server_terminals==0.4.4
jupyterlab==4.0.5
jupyterlab-pygments==0.2.2
jupyterlab_server==2.24.0
kiwisolver==1.4.4
lazy_loader==0.3
leidenalg==0.10.1
libmambapy @ file:///Users/runner/miniforge3/conda-bld/mamba-split_1680791351028/work/libmambapy
llvmlite==0.40.1
locket==1.0.0
mamba @ file:///Users/runner/miniforge3/conda-bld/mamba-split_1680791351028/work/mamba
markdown-it-py==3.0.0
MarkupSafe==2.1.3
matplotlib==3.7.2
matplotlib-inline==0.1.6
matplotlib-scalebar==0.8.1
mdurl==0.1.2
mistune==3.0.1
msgpack==1.0.5
multidict==6.0.4
multipledispatch==1.0.0
multiscale_spatial_image==0.11.2
natsort==8.4.0
nbclient==0.8.0
nbconvert==7.7.4
nbformat==5.9.2
nest-asyncio==1.5.7
networkx==3.1
notebook==7.0.2
notebook_shim==0.2.3
numba==0.57.1
numcodecs==0.11.0
numpy==1.23.4
ome-zarr==0.8.0
omnipath==1.0.7
overrides==7.4.0
packaging @ file:///home/conda/feedstock_root/build_artifacts/packaging_1681337016113/work
pandas==2.0.3
pandocfilters==1.5.0
param==1.13.0
parso==0.8.3
partd==1.4.0
patsy==0.5.3
```

```
pexpect==4.8.0
pickleshare==0.7.5
Pillow==10.0.0
PIMS==0.6.1
platformdirs==3.10.0
pluggy @ file:///home/conda/feedstock_root/build_artifacts/pluggy_1687776318736/work
prometheus-client==0.17.1
prompt-toolkit==3.0.39
psutil==5.9.5
ptyprocess==0.7.0
pure-eval==0.2.2
pyarrow==12.0.1
pyasn1==0.4.8
pycosat @ file:///Users/runner/miniforge3/conda-bld/pycosat_1666836623787/work
pycparser @ file:///home/conda/feedstock_root/build_artifacts/pycparser_1636257122734/work
pyct==0.5.0
pydantic==1.10.12
pygeos==0.14
Pygments==2.16.1
pynndescent==0.5.10
pyOpenSSL @ file:///home/conda/feedstock_root/build_artifacts/pyopenssl_1685514481738/work
pyparsing==3.0.9
pyproj==3.6.0
PySocks @ file:///home/conda/feedstock_root/build_artifacts/pysocks_1661604839144/work
python-dateutil @ file:///home/conda/feedstock_root/build_artifacts/python-dateutil_1626286286081/work
python-json-logger==2.0.7
pytz==2023.3
PyWavelets==1.4.1
PyYAML @ file:///Users/runner/miniforge3/conda-bld/pyyaml_1666772543719/work
pyzmq==25.1.1
referencing==0.30.2
requests @ file:///home/conda/feedstock_root/build_artifacts/requests_1684774241324/work
rfc3339-validator==0.1.4
rfc3986-validator==0.1.1
rich==13.5.2
rpds-py==0.9.2
rsa @ file:///home/conda/feedstock_root/build_artifacts/rsa_1614171254180/work
ruamel.yaml @ file:///Users/runner/miniforge3/conda-bld/ruamel.yaml_1686993977949/work
ruamel.yaml.clib @ file:///Users/runner/miniforge3/conda-bld/ruamel.yaml.clib_1670412881259/work
s3fs==2023.6.0
s3transfer @ file:///home/conda/feedstock_root/build_artifacts/s3transfer_1683241957497/work
scanpy==1.9.3
scikit-image==0.20.0
scikit-learn==1.3.0
scipy==1.11.2
seaborn==0.12.2
Send2Trash==1.8.2
session-info==1.0.0
shapely==2.0.1
six @ file:///home/conda/feedstock_root/build_artifacts/six_1620240208055/work
slicerator==1.1.0
sniffio==1.3.0
sortedcontainers==2.4.0
soupsieve==2.4.1
spatial_image==0.3.0
spatialdata==0.0.12
squidpy==1.3.0
stack-data==0.6.2
statsmodels==0.14.0
stdlib-list==0.9.0
tblib==2.0.0
terminado==0.17.1
texttable==1.6.7
threadpoolctl==3.2.0
tifffile==2023.8.12
tinycss2==1.2.1
tomli==2.0.1
toolz @ file:///home/conda/feedstock_root/build_artifacts/toolz_1657485559105/work
tornado==6.3.3
tqdm @ file:///home/conda/feedstock_root/build_artifacts/tqdm_1677948868469/work
traitlets==5.9.0
typing_extensions==4.5.0
tzdata==2023.3
umap-learn==0.5.3
uri-template==1.3.0
urllib3 @ file:///home/conda/feedstock_root/build_artifacts/urllib3_1678635778344/work
```

```
validators==0.21.2
wcwidth==0.2.6
webcolors==1.13
webencodings==0.5.1
websocket-client==1.6.2
wrapt==1.15.0
xarray==2022.12.0
xarray-dataclasses==1.6.0
xarray-datatree==0.0.12
xarray-schema==0.0.3
xarray-spatial==0.3.7
yarl==1.9.2
zarr==2.16.1
zict==3.0.0
zipp==3.16.2
zstandard==0.19.0
```

For manuscripts utilizing custom algorithms or software that are central to the research but not yet described in published literature, software must be made available to editors and reviewers. We strongly encourage code deposition in a community repository (e.g. GitHub). See the Nature Portfolio guidelines for submitting code & software for further information.

Data

Policy information about availability of data

All manuscripts must include a data availability statement. This statement should provide the following information, where applicable:

- Accession codes, unique identifiers, or web links for publicly available datasets
- A description of any restrictions on data availability
- For clinical datasets or third party data, please ensure that the statement adheres to our policy

The processed data for this project is available under GSE245311 under the url: https://www.ncbi.nlm.nih.gov/geo/query/acc.cgi?acc=GSE245311. The raw sequencing files are access restricted and can be accessed at the European Genome-Phenome Archive under the accession number: ____. The raw data for the mass cytometry experiments can be found under the flow repository ID FR-FCM-Z6S6 with the url: http://flowrepository.org/id/FR-FCM-Z6S6. Published counts data25 for reference mapping of the immune cells was downloaded from the url: https://atlas.fredhutch.org/data/nygc/multimodal/pbmc_multimodal.h5seurat. Published counts data2 for control human choroid plexus single-nucleus RNA-sequencing samples was downloaded under the accession number: GSE159812 under the url: http://www.ncbi.nlm.nih.gov/geo/query/acc.cgi?acc=GSE159812. Published counts38 data for reference mapping and comparative analyses for prenatal immune cells was downloaded under the url: https://github.com/linnarsson-lab/developing-human-brain/.
Published counts data43 for comparative analyses of the glioblastoma samples was downloaded from the urls: https://www.brainimmuneatlas.org/data_files/toDownload/filtered_feature_bc_matrix_HumanGBMciteSeq.zip and its metadata was found at the url: https://www.brainimmuneatlas.org/data_files/toDownload/annot_Human_TAM_DC_Mono_citeSeq.csv.

Human research participants

Policy information about studies involving human research participants and Sex and Gender in Research.

Reporting on sex and gender	Sex and gender were not disaggregated in the present study. Since the presented data is on cell identity it is expected to apply regardless of sex. The single-cell RNA-sequencing data contained 61 female and 41 male donors. The engraftment data contained only female patients.
Population characteristics	Limited information was available of the anonymized patients. The age range was between the 23rd gestational week and 79 years. The tissue from control patients were at least 2 cm from the pathological focus and radiologically and histologically normal. The glioblastoma samples were IDH-wildtype CNS grade 4 tumors in line with the WHO classification of CNS tumors.
Recruitment	The patient samples were prospectively recruited in accordance with the conditions outlined in the study protocol. Tissues that showed histological signs of pathology were excluded.
Ethics oversight	The study protocol was approved by the Ethics committee of the University of Freiburg Medical Center and local ethics committees (National Research Ethics Service in the United Kingdom and local committees associated with the National Institutes of Health bio banks).

Note that full information on the approval of the study protocol must also be provided in the manuscript.

Field-specific reporting

Please select the one below that is the best fit for your research. If you are not sure, read the appropriate sections before making your selection.

☒ Life sciences ☐ Behavioural & social sciences ☐ Ecological, evolutionary & environmental sciences

For a reference copy of the document with all sections, see nature.com/documents/nr-reporting-summary-flat.pdf

Life sciences study design

All studies must disclose on these points even when the disclosure is negative.

Sample size	The samples were acquired prospectively. No calculation of the sample size was performed. The sample size is in line with previous peer-reviewed studies (e.g. Movahedi et al, Nat Neurosci 2021; Friebel et al, Cell 2021; Klemm et al Cell 2021; Sankowski et al, Nat Neurosci 2019)
Data exclusions	To ensure robust data and minimize technical artifacts cells with unusual gene expression profiles were excluded. For single-cell and single-nucleus RNA-sequencing, cells with less than 500 and more than 4,000 detected genes and more than 20 percent mitochondrial transcripts were excluded. The presented CITE-Seq data only contains cells with more than 50 counts per cell. For Cel-Seq2 data, cells with less than 200 and more than 4,000 detected genes and more than 20 percent mitochondrial transcripts were excluded. Furthermore, clusters with biologically uninfomative low-quality cells were excluded after evaluation. The exclusion criterial were in line with previously applied strategies for Cel-Seq2 data. For Fixed RNA-profiling samples, cells with less than 50 and more than 1,000 detected genes were excluded. ISS data only contains cells with 5 or more counts per cell.
Replication	We have utilized 2 independent single-cell protocols and, where possible, included more than 3 patients per analyzed compartment. Additionally, the samples were validated at protein levels using CITE-Seq, mass cytometry and immunohistochemistry. The described findings were consistent across the methods and were comparable to published mouse datasets including Zeisel et al, Science 2015, van Hove et al, Nat Neurosci 2019; Jordão et al, Science 2019; Sankowski et al, EMBO J 2021.
Randomization	Randomization was not performed as the study does not include an intervention. Samples were collected prospectively and stratified according to the radiological and histological appearance as normal or pathological.
Blinding	Microscopic analyses were conducted by blinded experimenters. scRNA-Seq and mass spectrometry data were analyzed by algorithms in an unsupervised manner. Due to the unsupervised manner of the analysis, blinding is not expected to affect the results.

Reporting for specific materials, systems and methods

We require information from authors about some types of materials, experimental systems and methods used in many studies. Here, indicate whether each material, system or method listed is relevant to your study. If you are not sure if a list item applies to your research, read the appropriate section before selecting a response.

Materials & experimental systems

n/a	Involved in the study	
☐	☒	Antibodies
☒	☐	Eukaryotic cell lines
☒	☐	Palaeontology and archaeology
☒	☐	Animals and other organisms
☒	☐	Clinical data
☒	☐	Dual use research of concern

Methods

n/a	Involved in the study	
☒	☐	ChIP-seq
☐	☒	Flow cytometry
☒	☐	MRI-based neuroimaging

Antibodies

Antibodies used

Iba1 Rabbit (Rb) 1:500 Cat# 019-19741; Lot# CAF6806; Fujifilm Wako Pure Chemical Corporation, Osaka, Japan
Iba1 Guinea pig (Gp) 1:500 Cat# 234004; Lot# 1-1; Synaptic Systems Gesellschaft für neurobiologische Forschung, Entwicklung und Produktion mbH; Göttingen, Germany
Iba1 Rabbit 1:1,000 Cat # ab178846; Lot# GR207976-28; Abcam PLC, Cambridge, United Kingdom
CD206 Mouse (Ms) 1:500 Cat# H00004360-M02, Lot# Abnova; Taipei City, Taiwan
CD1c Mouse 1:100 Cat# ab156708; Lot# GR222523-49; Abcam PLC, Cambridge, United Kingdom
CD163 Rabbit 1:1,000 Cat# HPA04604-100UL; Lot# A107257; Sigma-Aldrich Chemie GmbH; Munich, Germany
S100A6 Rabbit 1:500 Cat# HPA007575-100UL; Lot# A80292; Sigma-Aldrich Chemie GmbH; Munich, Germany
SIGLEC1 Rabbit 1:200 Cat# HPA053457-100UL; Lot# A114014; Sigma-Aldrich Chemie GmbH; Munich, Germany
Collagen IV Goat (Gt) 1:100 Cat# AB769; Lot# 3296140; Sigma-Aldrich Chemie GmbH; Munich, Germany
GLUT5 (SLC2A5) Rabbit 1:250 Cat# HPA005449-100UL; Lot# A08479; Sigma-Aldrich Chemie GmbH; Munich, Germany
TMEM119 Rabbit 1:200 Cat # ab185333, Lot# GR3225698-1, Abcam PLC, Cambridge, United Kingdom
P2RY12 Rabbit 1:1,500 Cat # HPA014518-100ul, Lot# D118480; Sigma-Aldrich Chemie GmbH; Munich, Germany

Secondary antibodies
Alexa Fluor 488 (490/525) Donkey anti Goat 1:500 Cat# A11055; Lot# 2059218; Thermo Fisher Scientific Inc.; Waltham, USA
Alexa Fluor 568 (578/603) Donkey anti Rabbit 1:500 Cat# A10042; Lot# 2044343; Thermo Fisher Scientific Inc.; Waltham, USA
Alexa Fluor 647 (650/665) Donkey anti Mouse 1:500 Cat# A31571; Lot# 2045337; Thermo Fisher Scientific Inc.; Waltham, USA
Alexa Fluor 647 (650/665) Donkey anti Guinea pig 1:500 Cat# 706-605-148; Lot# 143565; Jackson ImmunoResearch Laboratories Inc.; West Grove, USA
DAPI (351/461) 1:10,000 Cat# 6335.2; Lot# 408275274; Carl Roth GmbH +Co. KG; Karlsruhe, Germany

FACS Antibodies

CD3 Mouse 1:100, clone SP34-2, Cat# 551916; Lot# B208643; BD Bioscience; Heidelberg, Germany;
CD11b Rabbit 1:800, clone M1/70, Cat# 101237; Lot# B245639; eBioscience Inc.; San Diego, USA
CD19 Mouse 1:100, clone SJ25C1, Cat# 363003; Lot# B275700; BioLegend; San Diego, USA
CD20 Mouse 1:400, clone 2H7, Cat# 302311; Lot# B257731; BioLegend; San Diego, USA
CD45 Mouse 1:100, clone HI30, Cat# 555485; Lot# 8012762; BD Bioscience; Heidelberg, Germany;
CD206 Mouse 1:400, clone 15-2, Cat# 321119; Lot# B278564; BioLegend; San Diego, USA
DAPI - 1:1,000 Cat# 6335.2; Lot# 408275274; Carl Roth GmbH +Co. KG; Karlsruhe, Germany
Human TruStain FcX - 1:200 Cat# 422302; Lot# B270787; BioLegend; San Diego, USA
NeuN (RBFOX3) Mouse 1:100, clone 1B7, Cat# NBP1-92693AF647; Lot# D109332; Novus Biological; Centennial, USA
Olig2 Mouse 1:100, clone 211F1.1, Cat# MABN50A4; Lot# GR3416724-2; Merck; Darmstadt, Germany

Validation

Immunohistochemistry antibodies were validated using positive and negative control stainings following the manufacturer's instructions. The antibodies were validated at different dilutions and pretreatments by experienced staff at the histology lab of the department. FACS antibodies were validated according to the manufacturer's instructions using all relevant isotype controls and fluorescence minus one controls.

Flow Cytometry

Plots

Confirm that:

☒ The axis labels state the marker and fluorochrome used (e.g. CD4-FITC).

☒ The axis scales are clearly visible. Include numbers along axes only for bottom left plot of group (a 'group' is an analysis of identical markers).

☒ All plots are contour plots with outliers or pseudocolor plots.

☒ A numerical value for number of cells or percentage (with statistics) is provided.

Methodology

Sample preparation

Samples were carefully homogenized using a potter in Hanks' Balanced Salt Solution (HBSS) containing 0.54% Glucose and 15mM 4-(2-hydroxyethyl)-1-piperazineethanesulfonic acid (HEPES) buffer. Enzymatic digestion was performed with 0.5 ml Accumax with shaking (800 rpm) at room temperature for 30 minutes. Afterwards, the suspension was filtered through a 70µm cell strainer and centrifuged at 375G for 5 minutes at 4 degrees. Then cells were either cryopreserved in fetal bovine serum:dimethylsufloxide (9:1), or directly washed with FACS buffer consisting of Dulbecco´s Phosphate Buffered Saline (DPBS) with 2 Vol.-% inactivated FCS and 1 mmol Ethylenediaminetetraacetic acid (EDTA). FC block was achieved using Human TruStain FcX in FACS-Buffer (1:200) for 20 minutes. For the 10X single-cell RNA-Seq protocol, cells were stained with anti-CD45 only. Samples analyzed with CITE-Seq were additionally incubated for 30 min with the dissolved antibody cocktail. mCEL-Seq2 samples were incubated with the antibody mastermix (CD3, CD11b, CD19, CD20, CD45, CD206) diluted in FACS-Buffer for 20 minutes. Afterwards the are washed with FACS-buffer and stained for viability with DAPI at a dilution of 1:1000 in FACS-Buffer. For the mCEL-Seq2 protocol, DAPI-Lin-CD45+CD206+ cells were sorted. For the 10X protocol, all CD45+ cells were sorted. For 10X single-cell protocols, DAPI-CD45 cells were sorted.

Single-nucleus suspensions were prepared using the Frankenstein community protocol (https://www.protocols.io/view/frankenstein-protocol-for-nuclei-isolation-from-f-5jyl8nx98l2w/v2 accessed on July 1st 2022).

Single-nucleus suspension preparation from FFPE tissue:
Nuclei were extracted from FFPE tissues using the demonstrated protocol supplied by the manufacturer (10X Genomics, CG000632, Rev A).

Instrument

Beckman Coulter MoFlo Astrios, 14 color (Lasers: 405 nm, 488 nm, 561 nm, 640 nm)
Becton Dickinson FACSAria III, 17 color (Lasers: 375/405 nm, 488 nm, 561 nm, 633 nm)

Software

FlowJo vX.0.7

Cell population abundance

Representative abundances of the analyzed DAPI-CD45+ and DAPI-Lin-CD45+CD206+ cells and of the DAPI+NeuN-OLIG2-nuclei are provided in Extended Data Figure 1

Gating strategy

For the mCEL-Seq2 protocol, DAPI-Lin-CD45+CD206+ cells were sorted. For the 10X protocol, all DAPI-CD45+ cells were sorted. Fpr single-nucleus mRNA sequencing DAPI+NeuN-Olig2- nuclei were sorted.

☒ Tick this box to confirm that a figure exemplifying the gating strategy is provided in the Supplementary Information.
